# A *Solanum neorickii* introgression population providing a powerful complement to the extensively characterized *Solanum pennellii* population

**DOI:** 10.1111/tpj.14095

**Published:** 2018-10-23

**Authors:** Yaacov Micha Brog, Sonia Osorio, Yoav Yichie, Saleh Alseekh, Elad Bensal, Andriy Kochevenko, Dani Zamir, Alisdair R. Fernie

**Affiliations:** ^1^ Faculty of Agriculture The Robert H. Smith Institute of Plant Sciences and Genetics in Agriculture at the Hebrew University of Jerusalem Rehovot 76100 Israel; ^2^ Department of Molecular Biology and Biochemistry Instituto de Hortofruticultura Subtropical y Mediterránea ‘La Mayora’ – University of Malaga – Consejo Superior de Investigaciones Científicas (IHSM‐UMA‐CSIC) Campus de Teatinos 29071 Málaga Spain; ^3^ Max‐Planck‐Institute of Molecular Plant Physiology Am Mühlenberg 1 14476 Potsdam‐Golm Germany; ^4^ Center of Plant Systems Biology and Biotechnology 4000 Plovdiv Bulgaria

**Keywords:** tomato, *Solanum lycopersicum*, *Solanum neorickii*, backcross inbred lines, high‐resolution mapping, phenylalanine, methionine

## Abstract

We present a complementary resource for trait fine‐mapping in tomato to those based on the intra‐specific cross between cultivated tomato and the wild tomato species *Solanum pennellii*, which have been extensively used for quantitative genetics in tomato over the last 20 years. The current population of backcross inbred lines (BILs) is composed of 107 lines derived after three backcrosses of progeny of the wild species *Solanum neorickii* (LA2133) and cultivated tomato (cultivar TA209) and is freely available to the scientific community. These *S. neorickii* BILs were genotyped using the 10K SolCAP single nucleotide polymorphism chip, and 3111 polymorphic markers were used to map recombination break points relative to the physical map of *Solanum lycopersicum*. The BILs harbor on average 4.3 introgressions per line, with a mean introgression length of 34.7 Mbp, allowing partitioning of the genome into 340 bins and thereby facilitating rapid trait mapping. We demonstrate the power of using this resource in comparison with archival data from the *S. pennellii* resources by carrying out metabolic quantitative trait locus analysis following gas chromatography–mass spectrometry on fruits harvested from the *S. neorickii* BILs. The metabolic candidate genes phenylalanine ammonia‐lyase and cystathionine gamma‐lyase were then tested and validated in F_2_ populations and via agroinfiltration‐based overexpression in order to exemplify the fidelity of this method in identifying the genes that drive tomato metabolic phenotypes.

## Significance statement

Here we present a complementary developed population derived from crossing the wild species *Solanum neorickii* (LA2133) with cultivated tomato (cultivar TA209) using sequenced backcrosses. The population is composed of 107 lines (backcross inbred lines, BILs) with 3111 polymorphic markers used to map recombination break points relative to the physical map of *Solanum lycopersicum*. In addition we carried out metabolic quantitative trait locus analysis on fruits harvested from the *S. neorickii* BILs. The metabolic candidate genes phenylalanine ammonia‐lyase and cystathionine gamma‐lyase were identified and validated.

## **Introduction**

The combination of a burgeoning human population and environmental deterioration means that we are faced with the need to find more effective solutions for feeding the planet (Ludewig and Sonnewald, 2016). To this end genetic improvement of crop plants represents a major approach to enhancing agricultural output. This is challenging, since natural genetic variance is commonly displayed across a quantitative phenotypic range as opposed to qualitative phenotypes that fall into discrete categories (Mackay, 2001; Fernie and Tohge, 2017). By contrast, for traits controlled by a single gene or several genes with large effects, variation in quantitative traits tends to be caused by segregation of multiple genes with individually small effects whose expression is shaped by interactions with other genes and the environment (Ofner *et al*., 2016).

The theoretical basis for the genetic dissection of complex traits is relatively simple. At a basic level, quantitative trait locus (QTL) mapping simply involves finding an association between a genetic marker and a measurable phenotype (Mauricio, 2001). Since the early research of Sax (1923), considerable effort has been expended to identify the genetic basis of continuous traits using linkage analysis. However, these pioneering works were severely constrained by the dependence on visible morphological markers (Barton and Keightley, 2002). Over the past three decades, the establishment of large collections of molecular and genetic markers which can be used to construct detailed genetic maps of both domesticated and experimental species has overcome these limitations (Doerge, 2002). Indeed such maps paved the way for modern‐day QTL mapping (Mackay, 2001; Mauricio, 2001; Doerge, 2002). Currently two major approaches are employed to genetically dissect complex traits: traditional QTL analyses that usually focus on bi‐parental populations (Salvi and Tuberosa, 2005) and genome‐wide association studies (GWAS; Atwell *et al*., 2010), which are now more commonly used.

As a general rule QTL experiments are designed with a limited number of sources of genetic variation in order to allow facile dissection of complex phenotypes. This can be accomplished by constructing a bi‐parental population originating from homozygous distantly related inbred lines that exhibit genetic polymorphism which drives phenotypic variation. In practice, several different crossing schemes are used to generate such experimental mapping populations. However, irrespective of the crossing scheme used, in all cases the parents are mated to generate an F_1_ population. There are several bi‐parental population structures that are widely used for the detection of QTLs. Recombinant inbred lines are created by self‐pollinating each of the F_2_ progeny for several consecutive generations (single seed descent). In the ‘F_2_ design’, the mapping population is generated by mating the F_1_ progeny to each other. By contrast in the ‘backcross design’, the mapping population is generated by crossing the F_1_ progeny to either, or both, of the parents. Several variations of the above‐mentioned crossing schemes have been designed to fully optimize the shuffling of parental alleles (Mauricio, 2001). For example, backcrossed inbred lines (BILs), near‐isogenic lines (NILs) and introgression lines (ILs) have all been demonstrated to facilitate the incorporation of desired alleles into an agriculturally superior genetic background (Tanksley and Nelson, 1996).

The use of traditional QTL mapping in experimental populations has become a standard procedure in quantitative genetics (Salvi and Tuberosa, 2005), but although thousands of research papers have reported original QTL data only a small proportion of these QTLs have been cloned (Nordborg and Weigel, 2008). Furthermore, those QTLs that have been cloned generally involve loci that exhibit large effects and high heritability (Salvi and Tuberosa, 2005). Following the release of the human genome sequence (Venter *et al*., 2001), GWAS have been extensively used to identify genes associated with an ever‐growing number of human diseases (Hindorff *et al*., 2009). In plants, GWAS studies have been successfully used to identify loci that explain a considerable proportion of phenotypic variation (Brachi *et al*., 2011). Genome‐wide association studies are similar to single marker mapping in that they both involve associating a single marker with a phenotype, albeit with a far greater power of detection due to the massive increase in the number of markers (which facilitates stronger associations). However, GWAS have several fundamental limitations; for example, they generate false positives due to population structure (Brachi *et al*., 2010) and often fail to detect minor‐frequency alleles or environment–genotype interactions, rendering the results of certain GWAS harder to reproduce (Greene *et al*., 2009). Statistical methods to control for population structure have been developed to reduce the increasing number of false‐positive associations in GWAS (Yu and Buckler, 2006); however, an alternative to this could be the complementary use of traditional linkage mapping. In the past few years a combination of these two approaches has been used to validate the position of QTLs in several studies in mice (Manenti *et al*., 2009), Arabidopsis (Brachi *et al*., 2010) and maize (Wen *et al*., 2014). These studies were among the first to point out the advantage of using combined approaches that link traditional mapping methods relying on recombination‐based genetic positions with high‐throughput genotyping techniques that enable dense molecular maps anchored to reference genomes (Sim *et al*., 2012). However, the use of dense molecular maps with thousands of markers with bi‐parental populations is limited by the fact that in order to fully exploit the potential provided by the high number of markers a growing number of individuals are needed to ensure that the requirement for a large number of recombination events is met. This allows higher resolution for mapping of the QTLs – but is non‐trivial given the high cost of the genotyping techniques and the amount of work involved in the generation, maintenance and extensive phenotyping of a large number of individuals.

Tomato (*Solanum lycopersicum*), belongs to the Solanaceae family which includes vegetable crops such as potato (*Solanum tuberosum*; Xu *et al*., 2011), eggplant (*Solanum melongena*; Hirakawa *et al*., 2014) and pepper (*Capsicum annuum*; Qin *et al*., 2014). The tomato genome is considered to be a reference for solanaceous species because it is one of the smallest diploid genomes within the Solanaceae family and a particularly high conservation of gene order (synteny) was recorded within the genus *Solanum* (Sato *et al*., 2012). It is also a model organism for the study of fleshy fruit development and ripening (Klee and Giovannoni, 2011), compound leaf development, floral systems and plant architecture (Kimura *et al*., 2008), as well as defense response(s)/resistance against abiotic and biotic stresses (Kennedy, 2003; Sun *et al*., 2011; Koenig *et al*., 2013). Moreover, due to the availability of robust phenotyping platforms for quantifying traits that determine agricultural output, tomato is an extremely useful system for studying multigenic complex traits (Gur *et al*., 2004). Furthermore, the broad germplasm comprising inter‐specific mapping populations (Tanksley and McCouch, 1997) and mutant populations (Menda *et al*., 2004) has proved to be a powerful tool for breaking down complex traits into their Mendelian components (see Rodriguez‐Leal *et al*., 2017; Soyk *et al*., 2017) – a challenging prerequisite for isolating complex trait genes.

**Figure 1:**
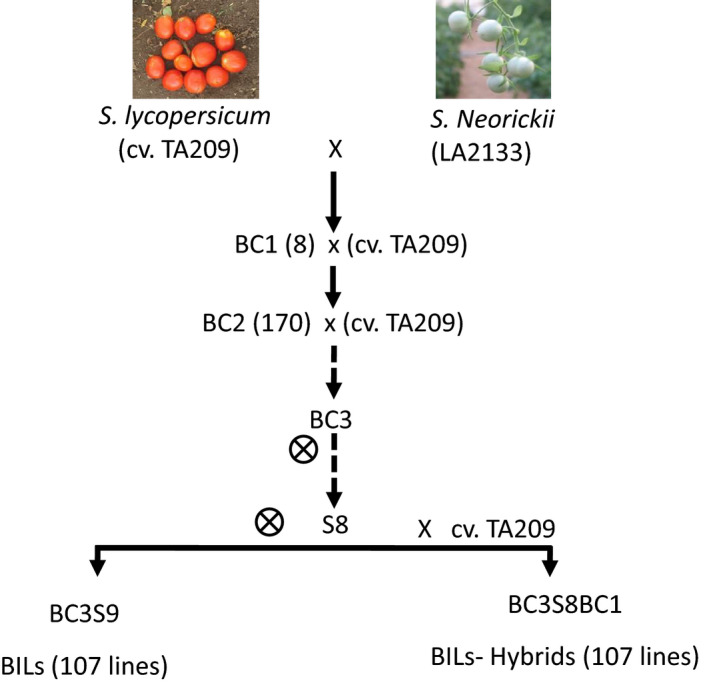
Breeding scheme of the backcross inbred lines (BILs). For the first cross, pollen was collected from *Solanum neorickii* and located on the stigma of cv. TA209 to produce the F_1_ plants. Advanced backcrosses with cv. TA209 were performed in order to minimize the *S. neorickii* genome introgressed in the BILs. The number of plants in each generation is shown in brackets. Successive selfing brought the population close to homozygosity. A final cross of the homozygous BILs with cv. TA209 generated a set of lines in a heterozygous condition (BILs‐hybrids).

Over the last 20 years one of the most studied inter‐specific tomato populations has been the *Solanum pennellii* IL population (Eshed and Zamir, 1995). The population comprises marker‐defined genomic regions taken from the green‐fruited wild species *S. pennellii* and introduced, through genetic crosses, onto the genetic background of the tomato inbred cv. M82 (Eshed and Zamir, 1995). The isogenic background of IL populations has made them suitable for the mapping of Mendelian phenotypes in tomato, and enabled the positional cloning of genes with major effects on distinct quantitative traits. Besides *Brix9‐2‐5*, which was one of the first QTLs subjected to positional cloning (Fridman *et al*., 2000, 2004), there are many examples of the use of the IL population for the dissection of qualitative and quantitative traits. Introgression lines have been publicly available and have been thoroughly phenotyped for hundreds of qualitative and quantitative traits, including repeated measurements of the same traits, thus allowing for the identification of more than 4000 QTLs. In recent years efforts have been made to produce hundreds of sub‐ILs for the IL population (Alseekh *et al*., 2013). This facilitates better mapping resolution, which alongside the presence of the sequenced parental genomes (Bolger *et al*., 2014) and the abundance of molecular markers (Sim *et al*., 2012) has already been useful in narrowing the size of the genomic region harboring QTLs (Gur *et al*., 2010). The recent release of an *S. pennellii* BIL population (Ofner *et al*., 2016) and the uptake of GWAS by tomato geneticists (Sauvage *et al*., 2014; Ye *et al*., 2017), have additionally extended the resources available for QTL mapping in tomato. Here we report on yet another genetic resource for tomato, namely a complementary BIL population generated from the cross between the wild tomato species *Solanum neorickii* and the tomato cv. TA209 (Figure 1). We illustrate the utility of this population by rapidly identifying a metabolite QTL for primary metabolites in the fruit. Two QTLs – those for phenylalanine on chromosome 10 and for methionine on chromosome 8 – were validated by the use of F_2_ families segregating for the trait in question and by agroinfiltration‐based overexpression studies.

## **Results**

### **Genome analysis of the** ***S. neorickii*** **BILs**

**Figure 2:**
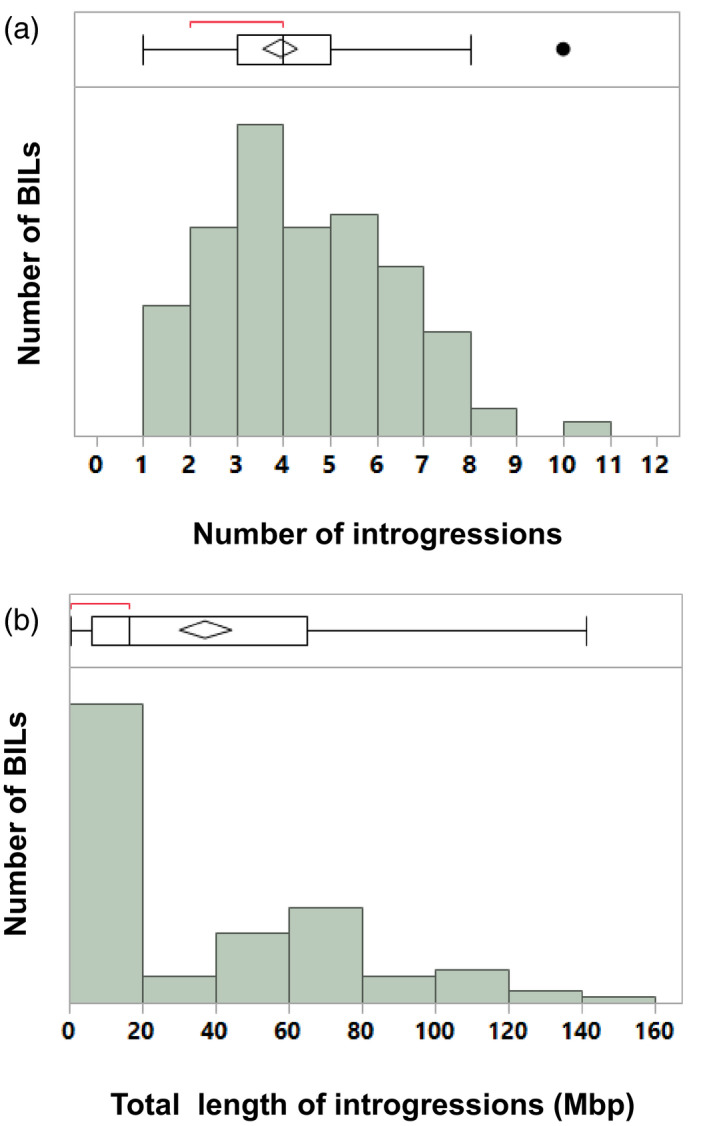
The number and length distribution of the backcross inbred line (BIL) introgressions. (a) The length distribution of introgressions. (b) Distribution of the number of introgressions in the BIL population. Box plots showing the mean and median length for all introgressions are presented.

Mapping analysis was carried out on the *S. neorickii* BILs based on 3111 single‐nucleotide polymorphism (SNP) markers that were polymorphic between the *S. neorickii* and *S. lycopersicum* parents (Figure S1, Table S1 in the online Supporting Information). The analysis revealed 34 out of 141 lines with no *S. neoricki* introgressions detected in *S. lycopersicum*. However, 107 lines exhibited at least a single introgression (Figure 2a), with the average number of introgressions per line being 4.3 and the most introgressions per line being 12 (Figure 2b). The size of each introgression was calculated based on the physical map of the tomato genome (v2.4). The margins of each line were identified by the markers that showed the *S. neorickii* SNPs and the following marker that showed the TA209 SNP (Table S1). The border of the introgression was arbitrarily calculated to be half‐way between the inclusive and exclusive wild‐species SNP. If the first or the last SNP on a chromosome showed a *S. neorickii* SNP then the tip of the chromosome was used for the length calculation. Based on these criteria, the mean sum of introgression length in the 107 lines was 37.3 Mbp while the median sum of introgression length per line was 16.3 Mbp.

### **Metabolite profiling of the** ***S. neorickii*** **BILs**

**Figure 3:**
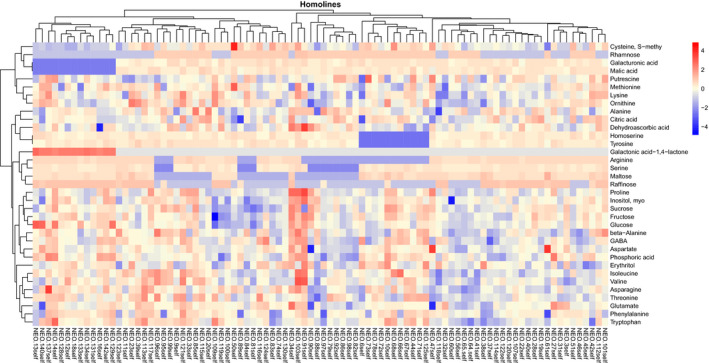
Heat map of the metabolite profiles of *Solanum neorickii* homozygous backcross inbred lines (BILs). Data are normalized to the average response calculated for the TA209 line. The scale is logarithmic. Values presented are means of three replicates and are shown in false‐color code.

We next subjected the 107 homozygous and 107 heterozygous BILs to GC‐MS‐based metabolite profiling (Schauer *et al*., 2006). For this purpose we took ripe pericarp material from fruit harvested from each homozygous line and its recurrent parent, *S. lycopersicum* TA209, in the harvests of 2008 and 2009 and heterozygous lines of the 2009 harvest and snap‐froze it in liquid nitrogen. We were able to determine the relative levels of 35 metabolites including sugars and sugar derivatives and organic and amino acids in every sample (values are presented in the heat maps of Figures 3 and S2 and Tables S2 and S3, respectively). The metabolites exhibited variance of between 0.05‐ and 133‐fold across the population, considerably larger ranges than those previously reported for *S. pennellii* and *S. chimielewskii* ILs (Schauer *et al*., 2006, 2008; Do *et al*., 2010).

However, given that each of these populations was based on a different cultivar of *S. lycopersicum* as recurrent parent it is currently impossible to ascertain if this difference is a consequence of the wild species introgressed or the cultivar which it is introgressed into. Irrespective of this, the differences observed here allowed the determination, using analysis of variance (anova), of a total of 71, 132 and 74 putative metabolite QTLs (mQTLs) in the 2008 and 2009 inbred lines and in the 2009 hybrid lines, respectively (Table S4). These corresponded to between 1 and 12 mQTLs per metabolite. However, only 25 of the mQTLs overlapped in at least two experiments (considering the homozygotes and heterozygotes as separate experiments). Interestingly, approximately 80% of these QTLs were similarly localized to previously identified ones (Schauer *et al*., 2006, 2008; Do *et al*., 2010), including those for amino acids such as isoleucine and lysine on chromosome 9 (Maloney *et al*., 2010; Kochevenko and Fernie, 2011) and sugars such as fructose and glucose on chromosome 6 (Pnueli *et al*., 1998). These QTLs were previously functionally tested as being due to polymorphism in enzymes of branched chain amino acid metabolism and/or cloned as the self‐prunning gene responsible for determinacy. By contrast, approximately 20% of the QTLs reported here are unique to this population. It will, ultimately, be very interesting to compare the number of QTLs that are found in the *S. neorickii* BILs but not the *S. pennellii* BILs; however, as yet, very few of the *S. pennellii* BILs have been subject to metabolite profiling. Indeed, to date, most of these have been associated with a region of chromosome 10 that was recently identified as a hotspot for metabolite canalization (Alseekh *et al*., 2017) In this region five QTLs were detected in the *S. neorickii* BILs and while six were detected in *S. pennellii* BILs. Interestingly, only two of the QTLs were conserved in this region, namely those for phenylalanine and galactinol, while three were unique to the *S. neorickii* BIL population, confirming our postulate that this material will be useful in its own right for identifying new QTLs. Given that they were strong QTLs and highly conserved in all three harvests (i.e. homozygous lines harvested in 2008 and 2009 and heterozygous lines harvested in 2009) we chose to carry out further validation studies on the mQTL for phenylalanine on chromosome 10 and that for methionine on chromosome 8.

**Figure 4:**
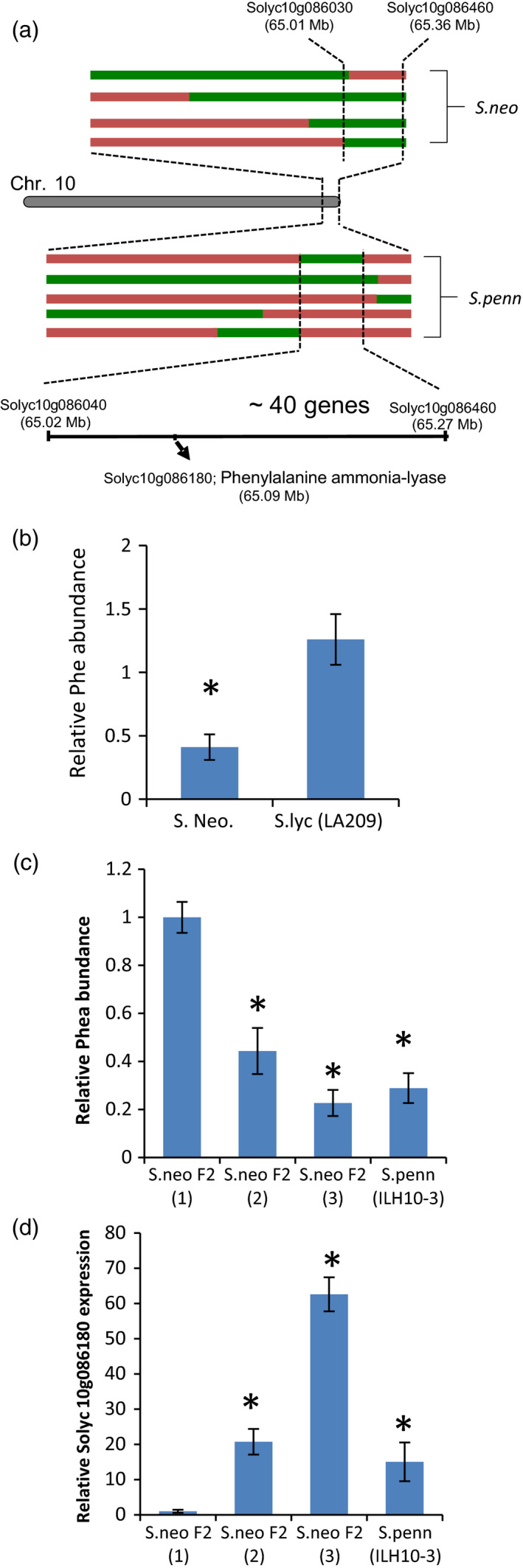
Fine mapping and validation of the phenylalanine quantitative trait loci (QTLs) on chromosome 10. (a) Fine mapping using the defining backcross inbred lines (BILs) of *Solanum neorickii* (s. neo) and *Solanum pennellii* (s. penn) for the phenylalanine QTL on chromosome 10. Green bars represent the wild introgression segments, red bars correspond to the cultivated background cv. M82 and TA209 segments. The schematic representation also shows the interval region (Mb) according to the SNP genomic position and the open reading frame of phenylalanine ammonia‐lyase gene. (b) Phenylalanine levels in *S. neorickii* BILs. Values represent the mean (± standard error) of three harvests. (c) Phenylalanine levels in F_2_ segregating *S. neorickii* families and the *S. pennellii* heterozygous introgression line ILH10‐3. The bar graph plots the relative content of phenylalanine in red fruit pericarp (± standard error). Numbers in parenthesis refer to (1) material homozygous for the cultivated tomato allele, (2) the heterozygote and (3) material homozygous for the wild species allele. (d) *Solyc10g086180* expression levels in fruit pericarp of F_2_ segregating *S. neorickii* families and the *S. pennellii* heterozygous introgression line ILH10‐3.

A corresponding QTL to the conserved mQTL for phenylalanine on chromosome 10 (Figure 4a) had previously been documented as localized to IL10‐3 of the *S. pennelli* IL population (Schauer *et al*., 2006). In the *S. neorickii* population, two QTLs for phenylalanine were identified. In the first QTL, mapped to chromosome 4 (Table S4), the wild *S. neorickii* allele increased the level of phenylalanine. The second QTL mapped to chromosome 10 and the wild allele resulted in a decrease in phenylalanine levels (Figure 4b, Table S4). The LOD (logarithm of odds) drop‐off interval around the corresponding genomic region spans more than 590 kbp and contains 88 annotated gene models. One of the annotated gene models in the corresponding genomic region is *Solyc10g086180*, which is functionally annotated as the key chorismate pathway enzyme phenylalanine ammonia‐lyase (PAL). In order to gain higher genetic resolution, we took advantage of recently available BIL populations derived from a cross between *S. pennellii* and the cultivated tomato (cv. M82; Ofner *et al*., 2016) to examine the levels of phenylalanine in selected BILs covering the QTL region. This experiment allowed us firstly to validate the QTL in a different population, and secondly to narrow down the QTL region to a 250‐kbp region containing 40 annotated gene models, including the previously described PAL gene (Figure 4a). In addition, and in order to validate the effect of the phenylalanine QTL from *S. neorickii*, seedlings obtained from the self‐pollinated heterozygous BILs neo‐089 and neo‐097 (F_2_ BILs) with an overlapping introgression in the investigated QTL region of chromosome 10 were tested in the field in 2013. Seedlings were genotyped prior to planting using cleaved amplified polymorphic sequence (CAPS) markers generated from the CT240 sequence which is physically located in the introgression region (Table S7). Tissue was collected from fruit pericarp of mature green and red fruits, mature and young leaves, and stems of the F_2_ families and the recurrent parent TA209, and subjected to GC‐MS analysis to determine the phenylalanine levels. A significant decrease in the relative content of phenylalanine was found in the red fruit pericarp when the mean values of plants homozygous for the wild‐species allele were compared with those of plants homozygous for the cultivated tomato allele (Figures 4c and S2). No significant difference in the phenylalanine levels was found in others tissues, indicating fruit specificity of this effect (Figure S3).

**Figure 5:**
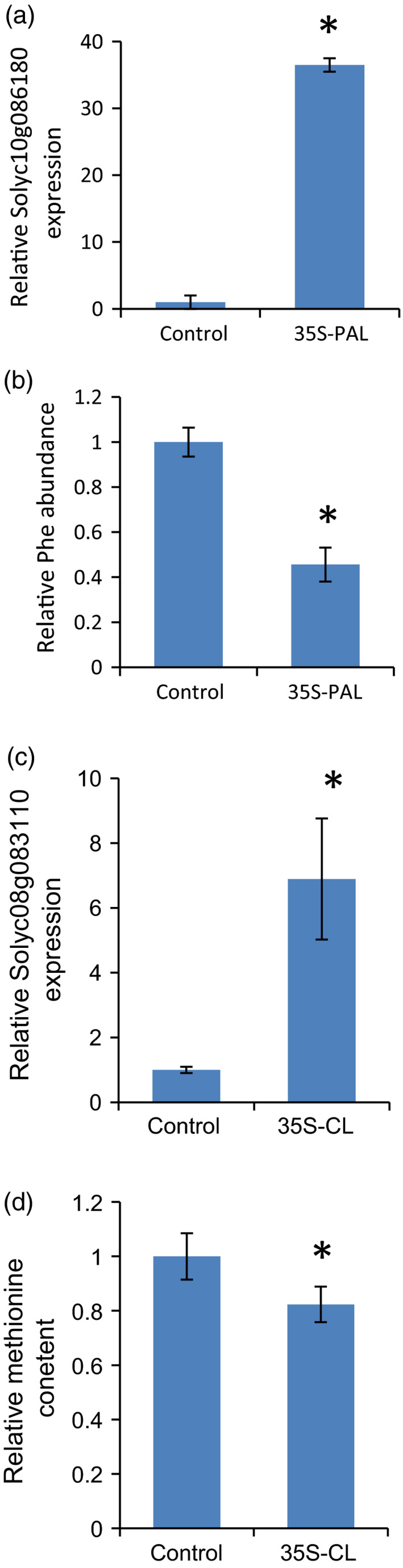
Transient overexpression of phenylalanine ammonia‐lyase (PAL) and cystathionine gamma‐lyase (CL). (a) Expression levels of PAL (*Solyc10g086180* gene) in fruit pericarp of lines transiently overexpressing an empty vector (control) or the 35S‐*Solyc10g086180* vector (35S‐PAL). Values presented the are mean of five biological replicates ± SD. Asterisks indicate significant differences from the control as assessed by Student's *t*‐test (*P* < 0.05). Data are from red ripe fruit which were inoculated at the breaker stage. (b) Phenylalanine levels in transient overexpression fruits of 35S‐PAL and the empty vector (control). Values presented are the mean of five biological replicates ± SD. Asterisks indicate significant differences from the control as assessed by Student's *t*‐test (*P* < 0.05). (c) Expression levels of CL (*Solyc08g083110* gene) in fruit pericarp of lines transiently overexpressing an empty vector (control) or the 35S‐*Solyc08g083110* vector (35S‐CL). Values presented the are mean of five biological replicates ± SD. Asterisks indicate significant differences from the control as assessed by Student's *t*‐test (*P* < 0.05). Data are from red ripe fruit which were inoculated at the breaker stage. (d) Methionine levels in transient overexpression fruits of 35S‐CL and the empty vector (control). Values presented are the mean of five biological replicates ± SD. Asterisks indicate significant differences from the control as assessed by Student's *t*‐test (*P* < 0.05). *P ≤ 0.05.

In a parallel approach, given that this was only 1 of 16 paralogs of PAL (*Solyc10g086180*) encoded by the tomato genome, we next evaluated, by quantitative (q)RT‐PCR, the expression of the 11 paralogs which displayed expression in tomato fruits (Sato *et al*., 2012; Bolger *et al*., 2014). For this purpose we analyzed mature green and red fruit of the recurrent parent and F_2_ families derived from neo89 and neo97 in addition to the heterozygote IL10‐3 (ILH10‐3). In red fruit, the expression of the gene encoded within this interval, *Soly10g086180*, was at least 20‐fold higher in all lines than in the recurrent parent, while expression in the F_2_ families having the genotype of the homozygote from wild species displayed expression levels which were over 60‐fold higher (Figure 4d). However, the levels of the transcript were unaltered in mature green fruits (Figure S3). Evaluation of the levels of phenylalanine, the substrate of this enzyme, revealed that it was dramatically decreased in all lines expressing wild alleles of PAL with respect to the recurrent parent (Figure 4c), confirming the candidature of this gene as a major determinant of phenylalanine levels in red tomato fruit. Intriguingly, analysis of the expression of the other 10 paralogs in mature green and red fruits revealed expression for only four further genes, but none of these genes had an expression level different from that of TA209 (Table 1). Similarly, the levels of phenylalanine were invariant in mature green fruit, stems and young and mature leaves of the various genotypes, suggesting that this effect is specific both to this paralog of PAL and to the red fruit (Figure S4). In addition, in order to provide additional support for the observed phenotypes, we performed genomic sequence analysis of the promoter regions (defined as 1000 bp upstream of the start codon) of the PAL gene and compared the promoter sequences by aligning the sequences from *S. lycopersicum* cv. M82 and *S. pennellii* genome sequences (Bolger *et al*., 2014). Results showed that there is a 21‐bp deletion in the M82 sequence at position −50 bp, while the coding region was very similar between M82 and *S. pennellii*, albeit there were a few SNPs (Figure S5). The *S. neorickii* allele showed a similar deletion in the promoter region to that of *S. pennellii*. Together, these results suggest that the wild alleles of this PAL gene harbored by both *S. neorickii* and *S. pennellii* resulted in elevated expression of the gene and thereby enhanced the efficiency of the PAL enzyme in metabolizing phenylalanine. To test this hypothesis we decided to carry out targeted reverse genetics using a transient overexpression strategy. Transient overexpression of genes in tomato fruit by agroinfiltration has been successfully used to investigate gene function (Voinnet *et al*., 2003). Injection of green fruits with a solution containing *Agrobacterium tumefaciens* harboring a pBI‐*Solyc10g086180* construct did not evoke any phenotypic changes in the fruit. Further analysis of *Solyc10g086180* transcripts by qRT‐PCR was performed in the pericarp of independent fruits that were injected with either the pBI‐*Solyc10g086180* construct or an empty vector control. These studies revealed a significant (more than 30‐fold) increase in expression in the pericarp of the fruits injected with the pBI‐*Solyc10 g086180* construct compared with the control (Figure 5a). To order to conclusively define *Solyc10g086180* as being responsible for the changes in phenylalanine levels, we next evaluated the phenylalanine content in the same fruits. As anticipated, a decrease of more than 50% in the levels of phenylalanine was confirmed in those fruits in which *Solyc10g086180* was overexpressed (Figure 5b).

**Table 1:** Expression of phenylalanine ammonia‐lyase (PAL) in mature green and red fruits of heterozygous and homozygous chromosomal segmental of F_2_ families from *Solanum neorikii* (*S. neo*), as well as the *Solanum pennellii* (*S. penne*) IL10‐3 heterozygous lines (ILH 10‐3). The abundance of PAL mRNAs was measured by quantitative RT‐PCR. Values are normalized to mean values of cv. TA209. Values are means ± SD of three to eight replicates depending of the genotype

Red fruit				
Gene ID	*S. neo* F_2_ (1)	*S. neo* F_2_ (2)	*S. neo* F_2_ (3)	*S. penne* (ILH 10‐3)
*Solyc09g007890*	1.01 ± 0.03	0.93 ± 0.08	1.11 ± 0.07	1.22 ± 0.11
*Solyc09g007900*	1.02 ± 0.05	1.07 ± 0.07	1.17 ± 0.08	1.16 ± 0.10
*Solyc09g007920*	0.97 ± 0.04	1.08 ± 0.06	1.17 ± 0.06	0.84 ± 0.07
*Solyc03g078270*	1.00 ± 0.03	1.05 ± 0.06	1.23 ± 0.07	1.26 ± 0.06

Mature green fruit				
Gene ID	TA209 (1)	neo89 × TA209 (2)	neo97 self (3)	ILH 10‐3
*Solyc09g007890*	1.04 ± 0.04	1.06 ± 0.06	1.17 ± 0.06	0.93 ± 0.04
*Solyc09g007900*	1.00 ± 0.04	1.2 ± 0.07	0.95 ± 0.06	0.85 ± 0.08
*Solyc09g007920*	0.98 ± 0.02	1.15 ± 0.04	0.88 ± 0.04	1.14 ± 0.06
*Solyc03g078270*	1.03 ± 0.06	1.22 ± 0.07	0.87 ± 0.04	0.92 ± 0.02

Numbers in parenthesis refer to: (1) material homozygous for the cultivated tomato allele, (2) the heterozygote and (3) material homozygous for the wild species allele.

**Figure 6:**
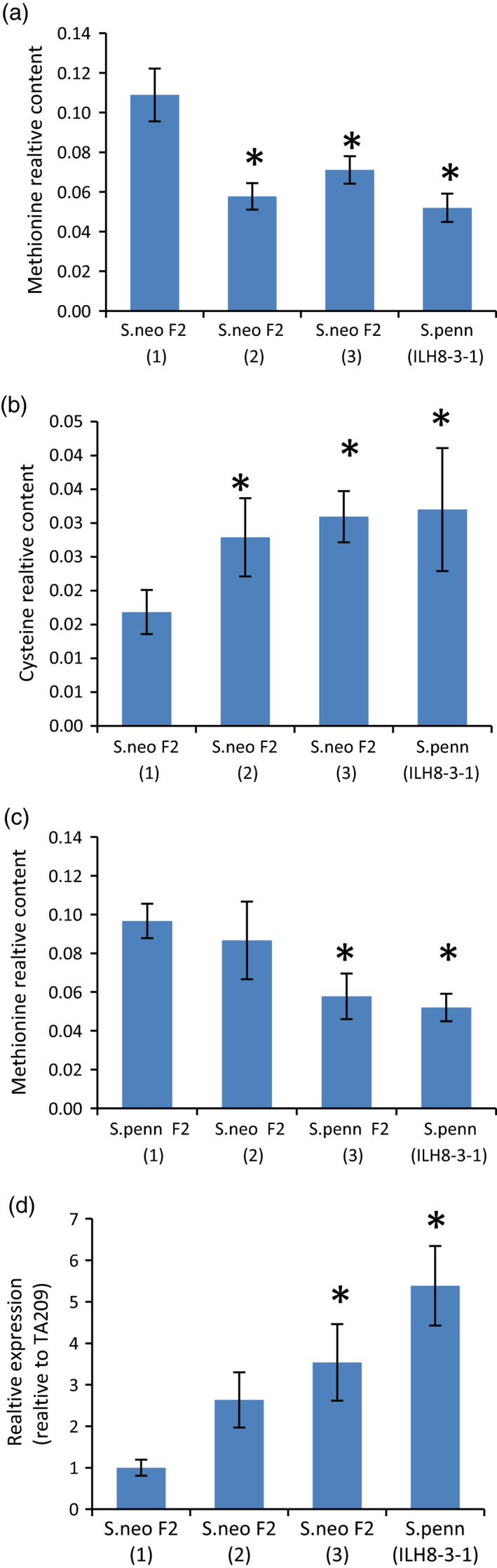
Levels of methionine and cysteine in *Solanum neorickii* and *Solanum pennellii* F_2_ families. Levels of methionine (a) and cysteine (b) in the red fruit pericarp of *S. neorickii* F_2_ segregating families and the *S. pennellii* heterozygous line (ILH8‐3‐1). (c) Levels of methionine in red fruit pericarp of *S. pennellii* F_2_ segregating families and the *S. pennellii* heterozygous line (ILH8‐3‐1). (d) Expression levels of the cystathionine gamma‐lyase gene (*Solyc08g083110*) in red fruit pericarp of *S. neorickii* F_2_ segregating families and the *S. pennellii* heterozygous line (ILH8‐3‐1). Bar graphs represent the relative content ± SE. Numbers in parenthesis refer to (1) material homozygous for the cultivated tomato allele, (2) the heterozygote and (3) material homozygous for the wild species allele. *P ≤ 0.05.

Similarly, a QTL corresponding to the conserved methionine mQTL has previously been documented to localize to IL8‐3‐1 of the *S. pennelli* IL population (Schauer *et al*., 2006). The QTL analysis using *S. neorickii* BILs identified several QTLs on different chromosomes (Table S4). For three of the identified QTLs, those on chromosomes 7, 8 and 11, the wild‐species allele resulted in a quantitative decrease of the trait, while for the QTL on chromosome 4 the wild allele resulted in a quantitative increase in methionine levels. The LOD drop‐off interval around the corresponding genomic region spans more than 537 kbp, containing 78 annotated gene models. One of the annotated genes is cystathionine gamma‐lyase which has a critical role in the methionine to cysteine pathway degrading cystathionine into cysteine and α‐ketobutyrate (Goyer *et al*., 2007). Next, in order to validate our finding, we measured the levels of methionine and cysteine in F_2_ segregating families derived from different *S. neorickii* and *S. pennellii* populations (Figure 6a–c). The results of these studies revealed a significantly lower methionine and higher cysteine content in F_2_ families harboring the wild allele, providing support for our hypothesized candidate gene. Furthermore, given that cystathionine gamma‐lyase (*Solyc08g083110*), the enzyme associated with methionine degradation, is encoded uniquely within the corresponding interval we carried out further experiments for this gene analogous to those described above. For this purpose we analyzed the expression of this gene in mature green and red ripe fruits of F_2_ families derived from the *S. neorickii* population alongside the heterozygote of IL8‐3‐1 (ILH 8‐3‐1). The expression of *Solyc08g083110* was significantly higher in both mature green fruit (Figure S6) and ripe red fruit (Figure 6d) in all lines bearing a wild‐species allele of this gene.

Injection of green fruits with a solution containing *Agrobacterium tumefaciens* harboring a pBI‐*Solyc08g083110* construct did not evoke any phenotypic changes in the fruit. Further analysis of *Solyc08g083110* transcripts by qRT‐PCR was performed in the pericarp of independent fruits that were injected with either the pBI‐*Solyc08g083110* construct or the empty vector. These studies revealed a more than sevenfold increase in expression in the pericarp of fruits injected with the pBI‐*Solyc08g083110* construct compared with the control (Figure 5c). In order to conclusively define *Solyc08g083110* as being responsible for the changes in methionine levels, we next evaluated the methionine and cysteine content in the same fruits. As anticipated, a mild yet significant decrease in the levels of methionine was confirmed in those fruits in which *Solyc08g083110* was overexpressed, but the levels of cysteine were invariant (Figures 5d and S9).

## **Discussion**

The *S. neorickii* population consists of 107 BILs that were genotyped with 3111 SNPs, covering all 12 tomato chromosomes and assembled according to cultivated tomato variety Heinz 1706 (Sato *et al*., 2012). The BILs divided the tomato genome into 340 mapping bins with an average of 4.3 introgressions per line, as described in Ofner *et al*. (2016)As such, the population structure does not offer as high a resolution as that recently described for the *S. pennellii* BILs for which 446 lines exist, and the population contains an average of 2.7 introgressions per line and 633 mapping bins. In spite of the lower resolution, as we describe in detail below, the *S. neorickii* BILs, like the *S. pennellii* BILs before them (Ofner *et al*., 2016), greatly shorten the time required for fine mapping and provide a more global resolution of wild‐species traits via the analysis of multiple independent lines carrying the introgressed segments. Furthermore, by contrast to the 600‐odd sub‐ILs that represent recombinants of particular segments but often lack high‐density maps of the recombinant events in question (Alseekh *et al*., 2013), the BILs are mapped to a high resolution. The utility of the *S. pennellii* BILs in providing higher resolution for the genetics underlying tomato traits is best illustrated by the large number of papers in which this resource was used. To date, this population has been used to identify five candidate genes involved in cuticular wax biosynthesis (Ofner *et al*., 2016), as well as genes encoding an enzyme in the biosynthetic pathway for insecticidal acyl sugar production (Ning *et al*., 2015; Fan *et al*., 2016) and in mapping traits for leaf complexity, leaflet shape, flowering time (Fulop *et al*., 2016), resistance to dodder (Krause *et al*., 2018) and metabolite canalization (Alseekh *et al*., 2017). The *S. pennellii* BILs were used to identify a homolog to the Arabidopsis gene *EID1* that is responsible for slowing down the circadian clock of cultivated tomato during domestication (Müller *et al*., 2015).

Here we analyzed mQTLs in the *S. neorickii* BILs. However, it is highly likely that this population will also provide an important resource for a wide range of other traits. The population was notably characterized by fewer conserved mQTLs than we determined previously for the *S. pennellii* (Schauer *et al*., 2006, 2008) and *Solanum chmielewskii* (Do *et al*., 2010) IL populations. Interestingly, some 80% of the conserved QTLs in the *S. neorickii* BILs were previously reported in either the *S. pennellii* or the *S. chmielewskii* IL populations, or both. Both those QTLs that are conserved on the introduction of diverse wild‐species alleles and those that are unique to the *S. neorickii* BILs are of interest; the former suggests that our contention that this population can be used as a powerful complementary resource to the *S. pennellii* introgression lines, particularly given the relatively small size of the population, largely holds true. By contrast, the fact that 20% of QTLs are new in this study suggests that there are some species‐specific differences that are likely to be very interesting to follow in future studies. Among the QTLs that were conserved are phenylalanine on chromosome 10 and methionine on chromosome 8. By contrast, the first reported GWAS study on primary metabolism in tomato did not reveal 44 candidate loci for fruit metabolic traits (Sauvage *et al*., 2014), and a larger study by the same group (Bauchet *et al*., 2017) did not identify associations with phenylalanine or methionine levels. This observation thus provides further support for the value of such populations. It will eventually be interesting to compare the QTL discovery efficiency of the *S. neorickii* and *S. pennellii* BIL populations; however, as mentioned above, from the small‐scale comparison that is already possibly it is clear that additional QTLs not found in the *S. pennellii* BIL population can be anticipated.

In this study we validated the quantitative importance of genes involved in the metabolism of both phenylalanine and methionine. In the case of the former a detailed evaluation revealed the following properties of the gene in question. First, alteration in its expression follows its expression pattern in *S. lycopersicum*, i.e. it is fruit specific, being mainly expressed in red ripe fruit and is more highly expressed in the wild species than the cultivated variety. Available expression data suggest that this specific gene is highly expressed in all wild species but that its expression is negligible in cultivated tomato (Koenig *et al*., 2013; Bolger *et al*., 2014). Similarly, expression QTL data (TFGD, http://ted.bti.cornell.edu/) show 80‐fold increased expression of PAL in *S. pennellii* IL10‐3 which harbors this gene. By comparison we found a 3.5‐fold increase in gene expression in the *S. neorickii* BILs. Available genome sequence data suggest that this is most likely due to a deletion in the promoter region of the cultivated tomato genome (Figure S5) which is not present in any of the wild‐species genomes sequenced to date (Aflitos *et al*., 2014). Consistent with these observations, a previous metabolomics survey of the wild species of the *S. lycopersicum* complex revealed that *S. neorickii*,* S. pennellii* and indeed all wild species studied exhibited considerably lower levels of phenylalanine than the cultivated tomato. Given that phenylalanine represents the precursor for a range of phenylpropanoids and volatiles it is not unreasonable to assume that reduction of this activity may have been selected for on domestication, since several of these compounds have a bitter taste. By contrast, certain volatiles have been identified to be highly beneficial to taste (Tieman *et al*., 2012, 2017; Rambla *et al*., 2014) and phenylpropanoids often confer both biotic and abiotic stress resistance (Tohge *et al*., 2016) as well as health benefits within the diet (Martin *et al*., 2013). Given that all of these are deficient in the cultivated tomato, reintroduction of a wild allele of this gene may prove a highly effective strategy for future crop improvement. In keeping with this theory it is interesting to note that IL10‐3 was additionally characterized by increased levels of several secondary metabolites, including hydroxycinnamates and flavonoids (Wen *et al*., 2015) which are formed downstream of the reaction catalyzed by PAL.

In the second example we were able to demonstrate that the methionine content of the fruit was quantitatively determined by the expression level of the cystathionine gamma‐lyase gene which encodes an enzyme in the pathway that converts methionine to cysteine. Interestingly, analysis of a segregating F_2_ population revealed that lines displaying a decreased level of methionine universally exhibited elevated levels of cysteine – as would be expected following the crossover theorem of Crabtree and Newsholme (1987), although admittedly the cysteine levels were not significantly changed in the transient overexpression experiments. Nevertheless, as was observed for the phenylalanine QTL, the level of expression of cystathionine gamma‐lyase, which is not fruit specific although it is predominantly expressed in fruits, was significantly higher in all green and red fruits containing a wild‐type allele while the methionine content was lower and, most convincingly, transient overexpression of this gene led to a decrease in the content of methionine and a decrease in the ratio of methionine to cysteine. The sequence polymorphism between M82 and *S. pennellii* revealed four insertions or deletions of different sizes, ranging between 6 and 18 bp, in the promoter region for cystathionine gamma‐lyase, while in addition four amino acids were deleted in M82 compared with the *S. pennellii* allele (Figures S7 and S8). Importantly, an additional amino acid residue is present in all wild species, suggesting a deletion upon cultivation. Similar to the case above for phenylalanine, the levels of methionine were significantly decreased in the fruits of all wild species (0.1 to 0.5 times the levels in cultivated M82; Schauer *et al*., 2005). Given that this reaction is not as well studied in tomato as that catalyzed by PAL, it will probably be highly informative to proceed to stable transformation to better analyze its *in vivo* functional role across a range of different tissues and developmental stages.

In addition to the availability of the germplasm described here and access to the genetic maps which describe it, all data reported here have been added to the Phenome Networks warehouse (http://phenome-networks.com/): raw data can be freely download the data analyzed within the database using a range of genetic and statistical software. Studies of a previously mapped QTLs are thus rendered far more facile in that the number of BILs needed to fine‐map the trait can be rapidly reduced. The process of gene identification is also further accelerated, as highlighted in the examples described above for the *S. pennellii* BILs. It is our firm belief that the *S. neorickii* population defined here will provide both a powerful complement to the *S. pennellii* BILs, and due to the new QTLs identified here will also be a potent tool for QTL mapping of the less well studied wild species *S. neorickii* in its own right.

## **Experimental Procedures**

### **Plant material**

The *S. neorickii* BILs were constructed from a cross between the green‐fruited, self‐compatible wild accession LA2133 and the processing‐tomato inbred variety cv. TA209 (*S. lycopersicum*) (Fulton *et al*., 2000).AUTHOR: Fulton *et al*., 2000 has not been included in the Reference List, please supply full publication details. F_1_ hybrids were then backcrossed for three cycles to the processing‐tomato inbred variety cv. TA209 as described by Fulton *et al*. (2000) followed by 10 cycles of self‐pollination in order to achieve BILs with maximum homozygosity of the wild genomic introgressions. The *S. neorickii* BILs are composed of a total of 142 lines; however, due to poor germination and problematic genotyping results, only 107 lines were analyzed in this work. In addition, hybrids for all BILs were produced in the background of the recurrent parent cv. TA209 in order to evaluate the wild introgressions in a heterozygous state.

### **Genotyping**

DNA was extracted from each of the *S. neorickii* BILs. Concentrations of 50–80 ng L^−1^ from each the *S. neorickii* BILs and the appropriate controls of LA2133, TA209 and their F_1_ hybrid were genotyped using an Illumina 10K SNP chip (https://www.illumina.com/). The genotyping was performed as described by Sim *et al*. (2012) in the Trait Genetics GmbH genotyping service (http://www.traitgenetics.com/). Primers and sequences of both sets of markers can be accessed at the Solanaceae Genome Networks (http://solgenomics.net/) database.

### ***Solanum neorickii*** **genetic map and quantitative trait locus mapping**

The genetic map was constructed from 3111 genome‐anchored SNP markers that were found to be polymorphic between the wild species *S. neorickii* and the recurrent parent cv. TA209. The markers were divided into 340 bins with an average length of 2.07 Mbp per bin and composited from an average of 9.15 SNPs per bin. The calculated map is 537.8 cM long and covers 91.5% of the wild‐species genome. Due to the incomplete genome coverage of the introgressions of the population there are more designated linkage groups than chromosomes, resulting in a total of 20 linkage groups. For example, chromosomes 1 and 3 are each composed of three linkage groups. The longest linkage group represents chromosome 4 (54.7 cM) and the shortest are mapped to chromosomes 1, 2, 3 and 11 (0 cM). These short linkage groups are in fact not true linkage groups, and each of them is composed of SNP markers that co‐segregate with no recombination events between them across the entire BIL population. Therefore, the corresponding genomic regions between and around these SNP markers are designated only on a physical scale (Mbp). The construction of genetic maps enabled the application of the Haley–Knott regression method that takes into consideration flanking markers to provide a better estimation of the presence of the QTL and its position.

### **Field trails**

The field experiments were performed in Akko, Israel. Seedlings were grown for 35 days in the Hishtil nursery in Ashkelon and then transplanted in the field with 50 cm between plants and 2 m between rows (one plant per m^2^). Whole‐genome surveys for BIL inbreds and hybrids were conducted for 2 years (2008 and 2009). Every year seedlings were transplanted in the field around the first week of April and harvested during the last week of July or the first week of August according to their relative maturation. Three biological triplicates were used for each line in each year.

The whole‐genome survey experiments where all BILs and BIL hybrids were evaluated were planted in a randomized block design

### **F**_**2**_ **validation**

After a preliminary whole‐genome survey using all BILs, seeds from self‐pollinated heterozygous BILs were collected and used the following year for QTL effect validation in a segregating F_2_ design. The respective genotypes in the F_2_ generation were classified by screening with PCR‐based DNA markers for the defined introgressed segment. In metabolite analysis samples from two independent families of the same genotype were considered as replicates.

### **Metabolite extraction for GC‐MS**

For primary metabolites an established GC‐time of flight (ToF)‐MS protocol was used. Plant material was extracted using a method described in Schauer *et al*. (2006). Metabolite profiling and data analysis were performed as described in Lisec *et al*. (2006) using a Chroma TOF 1.0 (Leco, https://www.leco.com/) and TagFinder 4.0 software and metabolites are reported following community‐based reporting standards (Table S5; Fernie *et al*., 2011).

### **Heat maps**

Heat maps were produced using the ‘heat map’ function of the R software. The log_2_‐transformed metabolite data from three biological replicates were used to create the heat maps.

### **Gene expression analysis**

Total RNA was extracted from fruit pericarp as described by Bugos *et al*. (1995) with minor changes. First‐strand cDNA synthesis of 500 mg of RNA in a final volume of 20 ml was performed with Moloney murine leukemia virus reverse transcriptase, Point Mutant RNase H Minus (Promega, http://www.promega.com/), according to the supplier's protocol using oligo(dT) T19 primer. Expression of the PAL genes was evaluated by qRT‐PCR using the fluorescent interacting dye SYBR Green in an iCycler detection system (Bio‐Rad, http://www.bio-rad.com/). Expression data were normalized to the reference genes elongation factor1a (Zanor *et al*., 2009) (GenBank accession no. X14449) and Ubiquitin3 (Osorio *et al*., 2012) (GenBank accession no. X58253). The primers used for qRT‐PCR analysis are listed in Table S6.

### **Transient overexpression of tomato fruit by agroinfiltration**

The full‐length cDNA of *Solyc10g086180* and *Solyc08g083110* genes from *S. neorickii* was cloned into the pBI121 vector (Figure S10). Transient overexpression was performed in *S. lycopersicum* cv. M82 fruits by the co‐infiltration of an *Agrobacterium tumefaciens* strain containing PBI‐S*olyc10g086180*, PBI‐*Solyc08g083110* and the pBin61‐*p19* construct to overexpress a gene encoding the silencing suppressor p19 protein of *Tomato bushy stunt virus* under the control of the CaMV 35S promoter (Zanor *et al*., 2009). For tomato fruit infiltration, flowers were labeled at anthesis and inoculated 3.5 weeks later (mature green stage) by agroinfiltrating 0.7 ml of bacterial suspension (a mix of the *A. tumefaciens* strain containing PBI‐S*olyc10g086180*, PBI‐*Solyc08g083110* and pBin61‐*p19* vectors in a 1:1:1 ratio) through the peduncle into the fruit. Agroinjected fruits were collected at breaker stage. Five fruits from the same position of four independent plants were agroinfiltrated. The agroinfiltration experiments were repeated three times.

## Conflicts of Interest

The authors declare no conflicts of interest.

## Supporting information


**Figure S1.** Graphical representation of the 107 *Solanum neorickii* backcross inbred lines.
**Figure S2.** Heat map of the metabolites profiles of *Solanum neorickii* homozygous backcross inbred line heterozygous lines.
**Figure S3.** Phenylalanine levels in mature green fruit, stem, mature and young leaves of F_2_ families from *Solanum neorickii*, as well as the *Solanum pennellii* heterozygous line (ILH10‐3).
**Figure S4** ***.** Solyc10g086180* expression in mature green fruit of F_2_ families from *Solanum neorickii*, as well as the *Solanum pennellii* heterozygous line (ILH10‐3).
**Figure S5**. Sequence analysis of 1000 bp upstream and the open reading frame of cv. M82 and *Solanum pennellii* for the phenylalanine ammonia‐lyase gene (*Solyc10g086180*).
**Figure S6** *. Solyc08g083110* expression in mature green fruit of F_2_ families from *Solanum neorickii*, as well as the *Solanum pennellii* heterozygous line (ILH8‐3‐1‐3).
**Figure S7.** Sequence analysis of 1000 bp upstream and the open reading frame of cv. M82 and *Solanum pennellii* for the cystathionine gamma lyase gene (*Solyc08g083110*).
**Figure S8.** Protein sequence of the cystathionine gamma lyase (*Solyc08g083110*) gene of cv. M82 and *S. pennellii*.
**Figure S9.** Cysteine levels in transient overexpression of the cystathionine gamma lyase (*Solyc08g083110*) gene and the control.
**Figure S10.** Vector map used for cloning and transient overexpression.

**Table S1.** Genotype data set used for 107 *Solanum neorickii* backcrossed introgression line populations.

**Table S2.** Average fold changes of the metabolite content of the homozygous backcrossed introgression lines compared with the parental control (cv. TA209) from 2008 to 2009.

**Table S3.** Fold changes of metabolite content of the heterozygous backcrossed introgression lines compared with the parental control (cv. TA209) from 2009.

**Table S4.** *Solanum neorickii* metabolic quantitative trait loci.

**Table S5.** Overview of the metabolite reporting list.

**Table S6.** Primers used for quantitative RT‐PCR analysis.

**Table S7.** Cleaved amplified polymorphic sequence markers used for genotyping the F_2_ generations from backcrossed introgression lines.

## References

[tpj14095-bib-0001] Aflitos, S. , Schijlen, E. , de Jong, H. ***et al.***; Tomato Genome Sequencing Consortium . (2014) Exploring genetic variation in the tomato (Solanum section Lycopersicon) clade by whole‐genome sequencing. Plant J. 80, 136–148. 25039268 10.1111/tpj.12616

[tpj14095-bib-0002] Alseekh, S. , Ofner, I. , Pleban, T. , Tripodi, P. , Di Dato, F. , Cammareri, M. , Mohammad, A. , Grandillo, S. , Fernie, A.R. and Zamir, D. (2013) Resolution by recombination: breaking up *Solanum pennellii* introgressions. Trends Plant Sci. 18, 536–538. 24029406 10.1016/j.tplants.2013.08.003

[tpj14095-bib-0003] Alseekh, S. , Tong, H. , Scossa, F. , Brotman, Y. , Vigroux, F. , Tohge, T. , Ofner, I. , Zamir, D. , Nikoloski, Z. and Fernie, A.R. (2017) Canalization of tomato fruit metabolism. Plant Cell, 29, 2753–2765. 29093214 10.1105/tpc.17.00367 PMC5728129

[tpj14095-bib-0004] Atwell, S. , Huang, Y.S. , Vilhjalmsson, B.J. ***et al.*** (2010) Genome‐wide association study of 107 phenotypes in *Arabidopsis thaliana* inbred lines. Nature, 465, 627–631. 20336072 10.1038/nature08800 PMC3023908

[tpj14095-bib-0005] Barton, N.H. and Keightley, P.D. (2002) Understanding quantitative genetic variation. Nat. Rev. Genet. 3, 11–21. 11823787 10.1038/nrg700

[tpj14095-bib-0006] Bauchet, G. , Grenier, S. , Samson, N. ***et al.*** (2017) Identification of major loci and genomic regions controlling acid and volatile content in tomato fruit: implications for flavor improvement. New Phytol. 215, 624–641. 28585324 10.1111/nph.14615

[tpj14095-bib-0007] Bolger, A. , Scossa, F. , Bolger, M.E. ***et al.*** (2014) The genome of the stress‐tolerant wild tomato species *Solanum pennellii* . Nat. Genet. 46, 1034–1038. 25064008 10.1038/ng.3046 PMC7036041

[tpj14095-bib-0008] Brachi, B. , Faure, N. , Horton, M. , Flahauw, E. , Vazquez, A. , Nordborg, M. , Bergelson, J. , Cuguen, J. and Roux, F. (2010) Linkage and association mapping of *Arabidopsis thaliana* flowering time in nature. PLoS Genet. 6, e1000940. 20463887 10.1371/journal.pgen.1000940 PMC2865524

[tpj14095-bib-0009] Brachi, B. , Morris, G.P. and Borevitz, J.O. (2011) Genome‐wide association studies in plants: the missing heritability is in the field. Genome Biol. 12, 232. 22035733 10.1186/gb-2011-12-10-232 PMC3333769

[tpj14095-bib-0010] Bugos, R.C. , Chiang, V.L. , Zhang, X.H. , Campbell, E.R. , Podila, G.K. and Campbell, W.H. (1995) RNA isolation from plant tissues recalcitrant to extraction in guanidine. Biotechniques, 19, 734–737. 8588907

[tpj14095-bib-0011] Crabtree, B. and Newsholme, E.A. (1987) A systematic approach to describing and analysing metabolic control systems. Trends Biochem. Sci. 12, 4.

[tpj14095-bib-0012] Do, P.T. , Prudent, M. , Sulpice, R. , Causse, M. and Fernie, A.R. (2010) The influence of fruit load on the tomato pericarp metabolome in a *Solanum chmielewskii* introgression line population. Plant Physiol. 154, 1128–1142. 20841452 10.1104/pp.110.163030 PMC2971594

[tpj14095-bib-0013] Doerge, R.W. (2002) Mapping and analysis of quantitative trait loci in experimental populations. Nat. Rev. Genet. 3, 43–52. 11823790 10.1038/nrg703

[tpj14095-bib-0014] Eshed, Y. and Zamir, D. (1995) An introgression line population of *Lycopersicon pennellii* in the cultivated tomato enables the identification and fine mapping of yield‐associated QTL. Genetics, 141, 1147–1162. 8582620 10.1093/genetics/141.3.1147 PMC1206837

[tpj14095-bib-0015] Fan, P.X. , Miller, A.M. , Schilmiller, A.L. , Liu, X.X. , Ofner, I. , Jones, A.D. , Zamir, D. and Last, R.L. (2016) In vitro reconstruction and analysis of evolutionary variation of the tomato acylsucrose metabolic network. Proc. Natl Acad. Sci. USA, 113, E239–E248. 26715757 10.1073/pnas.1517930113 PMC4720351

[tpj14095-bib-0016] Fernie, A.R. and Tohge, T. (2017) The genetics of plant metabolism. Annu. Rev. Genet. 51, 287–310. 10.1146/annurev-genet-120116-024640. 28876980

[tpj14095-bib-0017] Fernie, A.R. , Aharoni, A. , Willmitzer, L. , Stitt, M. , Tohge, T. , Kopka, J. , Carroll, A.J. , Saito, K. , Fraser, P.D. and DeLuca, V. (2011) Recommendations for reporting metabolite data. Plant Cell, 23, 2477–2482. 21771932 10.1105/tpc.111.086272 PMC3226225

[tpj14095-bib-0018] Fridman, E. , Pleban, T. and Zamir, D. (2000) A recombination hotspot delimits a wild‐species quantitative trait locus for tomato sugar content to 484 bp within an invertase gene. Proc. Natl Acad. Sci. USA, 97, 4718–4723. 10781077 10.1073/pnas.97.9.4718 PMC18299

[tpj14095-bib-0019] Fridman, E. , Carrari, F. , Liu, Y.S. , Fernie, A.R. and Zamir, D. (2004) Zooming in on a quantitative trait for tomato yield using interspecific introgressions. Science, 305, 1786–1789. 15375271 10.1126/science.1101666

[tpj14095-bib-0020] Fulop, D. , Ranjan, A. , Ofner, I. ***et al.*** (2016) A new advanced backcross tomato population enables high resolution leaf QTL mapping and gene identification. G3: Genes ‐ Genomes ‐ Genetics, 6, 3169–3184. 27510891 10.1534/g3.116.030536 PMC5068939

[tpj14095-bib-0021] Goyer, A. , Collakova, E. , Shachar‐Hill, Y. and Hanson, A.D. (2007) Functional characterization of a methionine gamma‐lyase in Arabidopsis and its implication in an alternative to the reverse trans‐sulfuration pathway. Plant Cell Physiol. 48, 232–242. 17169919 10.1093/pcp/pcl055

[tpj14095-bib-0022] Greene, C.S. , Penrod, N.M. , Williams, S.M. and Moore, J.H. (2009) Failure to replicate a genetic association may provide important clues about genetic architecture. PLoS ONE, 4, e5639. 19503614 10.1371/journal.pone.0005639 PMC2685469

[tpj14095-bib-0023] Gur, A. , Semel, Y. , Cahaner, A. and Zamir, D. (2004) Real time QTL of complex phenotypes in tomato interspecific introgression lines. Trends Plant Sci. 9, 107–109. 15058274 10.1016/j.tplants.2004.01.003

[tpj14095-bib-0024] Gur, A. , Osorio, S. , Fridman, E. , Fernie, A.R. and Zamir, D. (2010) hi2‐1, a QTL which improves harvest index, earliness and alters metabolite accumulation of processing tomatoes. Theor. Appl. Genet. 121, 1587–1599. 20680612 10.1007/s00122-010-1412-8 PMC2963733

[tpj14095-bib-0025] Hindorff, L.A. , Sethupathy, P. , Junkins, H.A. , Ramos, E.M. , Mehta, J.P. , Collins, F.S. and Manolio, T.A. (2009) Potential etiologic and functional implications of genome‐wide association loci for human diseases and traits. Proc. Natl Acad. Sci. USA, 106, 9362–9367. 19474294 10.1073/pnas.0903103106 PMC2687147

[tpj14095-bib-0026] Hirakawa, H. , Shirasawa, K. , Miyatake, K. ***et al.*** (2014) Draft genome sequence of eggplant (*Solanum melongena* L.): the representative solanum species indigenous to the old world. DNA Res. 21, 649–660. 25233906 10.1093/dnares/dsu027 PMC4263298

[tpj14095-bib-0027] Kennedy, G.G. (2003) Tomato, pests, parasitoids, and predators: tritrophic interactions involving the genus Lycopersicon. Annu. Rev. Entomol. 48, 51–72. 12194909 10.1146/annurev.ento.48.091801.112733

[tpj14095-bib-0028] Kimura, S. , Koenig, D. , Kang, J. , Yoong, F.Y. and Sinha, N. (2008) Natural variation in leaf morphology results from mutation of a novel KNOX gene. Curr. Biol. 18, 672–677. 18424140 10.1016/j.cub.2008.04.008

[tpj14095-bib-0029] Klee, H.J. and Giovannoni, J.J. (2011) Genetics and control of tomato fruit ripening and quality attributes In Annual Review of Genetics, Vol 45 (Bassler B.L., Lichten M. and Schupbach G., eds). Palo Alto, CA: Annual Reviews, pp. 41–59. 10.1146/annurev-genet-110410-132507 22060040

[tpj14095-bib-0030] Kochevenko, A. and Fernie, A.R. (2011) The genetic architecture of branched‐chain amino acid accumulation in tomato fruits. J. Exp. Bot. 62, 3895–3906. 21436187 10.1093/jxb/err091 PMC3134350

[tpj14095-bib-0031] Koenig, D. , Jimenez‐Gomez, J.M. , Kimura, S. ***et al.*** (2013) Comparative transcriptomics reveals patterns of selection in domesticated and wild tomato. Proc. Natl Acad. Sci. USA, 110, E2655–E2662. 23803858 10.1073/pnas.1309606110 PMC3710864

[tpj14095-bib-0032] Krause, K. , Johnsen, H.R. , Pielach, A. , Lund, L. , Fischer, K. and Rose, J.K.C. (2018) Identification of tomato introgression lines with enhanced susceptibility or resistance to infection by parasitic giant dodder (*Cuscuta reflexa*). Physiol. Plant. 162, 205–218. 29080211 10.1111/ppl.12660

[tpj14095-bib-0033] Lisec, J. , Schauer, N. , Kopka, J. , Willmitzer, L. and Fernie, A.R. (2006) Gas chromatography mass spectrometry‐based metabolite profiling in plants. Nat. Protoc. 1, 387–396. 17406261 10.1038/nprot.2006.59

[tpj14095-bib-0034] Ludewig, F. and Sonnewald, U. (2016) Demand for food as driver for plant sink development. J. Plant Physiol. 203, 110–115. 27316916 10.1016/j.jplph.2016.06.002

[tpj14095-bib-0035] Mackay, T.F.C. (2001) The genetic architecture of quantitative traits. Annu. Rev. Genet. 35, 303–339. 11700286 10.1146/annurev.genet.35.102401.090633

[tpj14095-bib-0036] Maloney, G.S. , Kochevenko, A. , Tieman, D.M. , Tohge, T. , Krieger, U. , Zamir, D. , Taylor, M.G. , Fernie, A.R. and Klee, H.J. (2010) Characterization of the branched‐chain amino acid aminotransferase enzyme family in tomato. Plant Physiol. 153, 925–936. 20435740 10.1104/pp.110.154922 PMC2899903

[tpj14095-bib-0037] Manenti, G. , Galvan, A. , Pettinicchio, A. , Trincucci, G. , Spada, E. , Zolin, A. , Milani, S. , Gonzalez‐Neira, A. and Dragani, T.A. (2009) mouse genome‐wide association mapping needs linkage analysis to avoid false‐positive loci. PLoS Genet. 5, e1000331. 19132132 10.1371/journal.pgen.1000331 PMC2614123

[tpj14095-bib-0038] Martin, C. , Zhang, Y. , Tonelli, C. and Petroni, K. (2013) Plants, diet, and health In Annual Review of Plant Biology, Vol 64 (Merchant S.S. ed). Palo Alto, CA: Annual Review Publishers, pp. 19–46. 10.1146/annurev-arplant-050312-120142 23451785

[tpj14095-bib-0039] Mauricio, R. (2001) Mapping quantitative trait loci in plants: uses and caveats for evolutionary biology. Nat. Rev. Genet. 2, 370–381. 11331903 10.1038/35072085

[tpj14095-bib-0040] Menda, N. , Semel, Y. , Peled, D. , Eshed, Y. and Zamir, D. (2004) In silico screening of a saturated mutation library of tomato. Plant J. 38, 861–872. 15144386 10.1111/j.1365-313X.2004.02088.x

[tpj14095-bib-0041] Müller, N.A. , Wijnen, C.L. , Srinivasan, A. ***et al.*** (2015) Domestication selected for deceleration of the circadian clock in cultivated tomato. Nat. Genet. 48, 89. 26569124 10.1038/ng.3447

[tpj14095-bib-0042] Ning, J. , Moghe, G.D. , Leong, B. , Kim, J. , Ofner, I. , Wang, Z.Z. , Adams, C. , Jones, A.D. , Zamir, D. and Last, R.L. (2015) A feedback‐insensitive isopropylmalate synthase affects acylsugar composition in cultivated and wild tomato. Plant Physiol. 169, 1821–1835. 25986128 10.1104/pp.15.00474 PMC4634047

[tpj14095-bib-0043] Nordborg, M. and Weigel, D. (2008) Next‐generation genetics in plants. Nature, 456, 720–723. 19079047 10.1038/nature07629

[tpj14095-bib-0044] Ofner, I. , Lashbrooke, J. , Pleban, T. , Aharoni, A. and Zamir, D. (2016) *Solanum pennellii* backcross inbred lines (BILs) link small genomic bins with tomato traits. Plant J. 87, 151–160. 27121752 10.1111/tpj.13194

[tpj14095-bib-0045] Osorio, S. , Alba, R. , Nikoloski, Z. , Kochevenko, A. , Fernie, A.R. and Giovannoni, J.J. (2012) Integrative comparative analyses of transcript and metabolite profiles from pepper and tomato ripening and development stages uncovers species‐specific patterns of network regulatory behavior. Plant Physiol. 159, 1713–1729. 22685169 10.1104/pp.112.199711 PMC3425208

[tpj14095-bib-0046] Pnueli, L. , Carmel‐Goren, L. , Hareven, D. , Gutfinger, T. , Alvarez, J. , Ganal, M. , Zamir, D. and Lifschitz, E. (1998) The SELF‐PRUNING gene of tomato regulates vegetative to reproductive switching of sympodial meristems and is the ortholog of CEN and TFL1. Development, 125, 1979–1989. 9570763 10.1242/dev.125.11.1979

[tpj14095-bib-0047] Qin, C. , Yu, C.S. , Shen, Y.O. ***et al.*** (2014) Whole‐genome sequencing of cultivated and wild peppers provides insights into Capsicum domestication and specialization. Proc. Natl Acad. Sci. USA, 111, 5135–5140. 24591624 10.1073/pnas.1400975111 PMC3986200

[tpj14095-bib-0048] Rambla, J.L. , Tikunov, Y.M. , Monforte, A.J. , Bovy, A.G. and Granell, A. (2014) The expanded tomato fruit volatile landscape. J. Exp. Bot. 65, 4613–4623. 24692651 10.1093/jxb/eru128

[tpj14095-bib-0049] Rodriguez‐Leal, D. , Lemmon, Z.H. , Man, J. , Bartlett, M.E. and Lippman, Z.B. (2017) Engineering quantitative trait variation for crop improvement by genome editing. Cell, 171, 470–480.e8. 28919077 10.1016/j.cell.2017.08.030

[tpj14095-bib-0050] Salvi, S. and Tuberosa, R. (2005) To clone or not to clone plant QTLs: present and future challenges. Trends Plant Sci. 10, 297–304. 15949764 10.1016/j.tplants.2005.04.008

[tpj14095-bib-0051] Sato, S. , Tabata, S. , Hirakawa, H. ***et al.***; Tomato Genome Consortium . (2012) The tomato genome sequence provides insights into fleshy fruit evolution. Nature, 485, 635–641. 22660326 10.1038/nature11119 PMC3378239

[tpj14095-bib-0052] Sauvage, C. , Segura, V. , Bauchet, G. , Stevens, R. , Do, P.T. , Nikoloski, Z. , Fernie, A.R. and Causse, M. (2014) Genome‐wide association in tomato reveals 44 candidate loci for fruit metabolic traits. Plant Physiol. 165, 1120–1132. 24894148 10.1104/pp.114.241521 PMC4081326

[tpj14095-bib-0053] Sax, K. (1923) The association of size differences with seed‐coat pattern and pigmentation in PHASEOLUS VULGARIS. Genetics, 8, 552–558. 17246026 10.1093/genetics/8.6.552 PMC1200765

[tpj14095-bib-0054] Schauer, N. , Zamir, D. and Fernie, A.R. (2005) Metabolic profiling of leaves and fruit of wild species tomato: a survey of the *Solanum lycopersicum* complex. J. Exp. Bot. 56, 297–307. 15596477 10.1093/jxb/eri057

[tpj14095-bib-0055] Schauer, N. , Semel, Y. , Roessner, U. ***et al.*** (2006) Comprehensive metabolic profiling and phenotyping of interspecific introgression lines for tomato improvement. Nat. Biotechnol. 24, 447–454. 16531992 10.1038/nbt1192

[tpj14095-bib-0056] Schauer, N. , Semel, Y. , Balbo, I. , Steinfath, M. , Repsilber, D. , Selbig, J. , Pleban, T. , Zamir, D. and Fernie, A.R. (2008) Mode of inheritance of primary metabolic traits in tomato. Plant Cell, 20, 509–523. 18364465 10.1105/tpc.107.056523 PMC2329927

[tpj14095-bib-0057] Sim, S.C. , Durstewitz, G. , Plieske, J. ***et al.*** (2012) Development of a large SNP genotyping array and generation of high‐density genetic maps in tomato. PLoS ONE, 7, e40563. 22802968 10.1371/journal.pone.0040563 PMC3393668

[tpj14095-bib-0058] Soyk, S. , Lemmon, Z.H. , Oved, M. ***et al.*** (2017) Bypassing negative epistasis on yield in tomato imposed by a domestication gene. Cell, 169, 1142–1155.e12. 28528644 10.1016/j.cell.2017.04.032

[tpj14095-bib-0059] Sun, J.Q. , Jiang, H.L. and Li, C.Y. (2011) Systemin/Jasmonate‐mediated systemic defense signaling in tomato. Mol. Plant, 4, 607–615. 21357647 10.1093/mp/ssr008

[tpj14095-bib-0060] Tanksley, S.D. and McCouch, S.R. (1997) Seed banks and molecular maps: unlocking genetic potential from the wild. Science, 277, 1063–1066. 9262467 10.1126/science.277.5329.1063

[tpj14095-bib-0061] Tanksley, S.D. and Nelson, J.C. (1996) Advanced backcross QTL analysis: a method for the simultaneous discovery and transfer of valuable QTLs from unadapted germplasm into elite breeding lines. Theor. Appl. Genet. 92, 191–203. 24166168 10.1007/BF00223376

[tpj14095-bib-0062] Tieman, D. , Bliss, P. , McIntyre, L.M. ***et al.*** (2012) The chemical interactions underlying tomato flavor preferences. Curr. Biol. 22, 1035–1039. 22633806 10.1016/j.cub.2012.04.016

[tpj14095-bib-0063] Tieman, D. , Zhu, G. , Resende, M.F. Jr ***et al.*** (2017) A chemical genetic roadmap to improved tomato flavor. Science, 355, 391–394. 28126817 10.1126/science.aal1556

[tpj14095-bib-0064] Tohge, T. , Wendenburg, R. , Ishihara, H. ***et al.*** (2016) Characterization of a recently evolved flavonol‐phenylacyltransferase gene provides signatures of natural light selection in Brassicaceae. Nat. Commun. 7, 12399. 27545969 10.1038/ncomms12399 PMC4996938

[tpj14095-bib-0065] Venter, J.C. , Adams, M.D. , Myers, E.W. ***et al.*** (2001) The sequence of the human genome. Science, 291, 1304–1351. 11181995 10.1126/science.1058040

[tpj14095-bib-0066] Voinnet, O. , Rivas, S. , Mestre, P. and Baulcombe, D. (2003) An enhanced transient expression system in plants based on suppression of gene silencing by the p19 protein of tomato bushy stunt virus (Retracted article. See vol. 84, pg. 846, 2015). Plant J. 33, 949–956. 12609035 10.1046/j.1365-313x.2003.01676.x

[tpj14095-bib-0067] Wen, W.W. , Li, D. , Li, X. ***et al.*** (2014) Metabolome‐based genome‐wide association study of maize kernel leads to novel biochemical insights. Nat. Commun. 5, 3438. 24633423 10.1038/ncomms4438 PMC3959190

[tpj14095-bib-0068] Wen, W. , Li, K. , Alseekh, S. ***et al.*** (2015) Genetic determinants of the network of primary metabolism and their relationships to plant performance in a maize recombinant inbred line population. Plant Cell, 27, 1839–1856. 26187921 10.1105/tpc.15.00208 PMC4531352

[tpj14095-bib-0069] Xu, X. , Pan, S.K. , Cheng, S.F. ***et al.***; Potato Genome Sequencing Consortium . (2011) Genome sequence and analysis of the tuber crop potato. Nature, 475, 189–195. 21743474 10.1038/nature10158

[tpj14095-bib-0070] Ye, J. , Wang, X. , Hu, T.X. ***et al.*** (2017) An InDel in the promoter of Al‐ACTIVATED MALATE TRANSPORTER9 selected during tomato domestication determines fruit malate contents and aluminum tolerance. Plant Cell, 29, 2249–2268. 28814642 10.1105/tpc.17.00211 PMC5635988

[tpj14095-bib-0071] Yu, J.M. and Buckler, E.S. (2006) Genetic association mapping and genome organization of maize. Curr. Opin. Biotechnol. 17, 155–160. 16504497 10.1016/j.copbio.2006.02.003

[tpj14095-bib-0072] Zanor, M.I. , Osorio, S. , Nunes‐Nesi, A. ***et al.*** (2009) RNA interference of LIN5 in tomato confirms its role in controlling Brix content, uncovers the influence of sugars on the levels of fruit hormones, and demonstrates the importance of sucrose cleavage for normal fruit development and fertility. Plant Physiol. 150, 1204–1218. 19439574 10.1104/pp.109.136598 PMC2705052

