# Supplementary material for: A Solanum neorickii introgression population providing a powerful complement to the extensively characterized Solanum pennellii population
Source: Plant J. 2018 Oct 23;97(2):391–403. doi: 10.1111/tpj.14095 (PMC7379295; doi:10.1111/tpj.14095)
Supplement: Supplementary file 1 — Figure S1. Graphical representation of the 107 Solanum neorickii backcross inbred lines. Figure S2. Heat map of the metabolites profiles of Solanum neorickii homozygous backcross inbred line heterozygous lines. Figure S3. Phenylalanine levels in mature green fruit, stem, mature and young leaves of F2 families from Solanum neorickii, as well as the Solanum pennellii heterozygous line (ILH10‐3). Figure S4 . Solyc10g086180 expression in mature green fruit of F2 families from Solanum neorickii, as well as the Solanum pennellii heterozygous line (ILH10‐3). Figure S5. Sequence analysis of 1000 bp upstream and the open reading frame of cv. M82 and Solanum pennellii for the phenylalanine ammonia‐lyase gene (Solyc10g086180). Figure S6 . Solyc08g083110 expression in mature green fruit of F2 families from Solanum neorickii, as well as the Solanum pennellii heterozygous line (ILH8‐3‐1‐3). Figure S7. Sequence analysis of 1000 bp upstream and the open reading frame of cv. M82 and Solanum pennellii for the cystathionine gamma lyase gene (Solyc08g083110). Figure S8. Protein sequence of the cystathionine gamma lyase (Solyc08g083110) gene of cv. M82 and S. pennellii. Figure S9. Cysteine levels in transient overexpression of the cystathionine gamma lyase (Solyc08g083110) gene and the control. Figure S10. Vector map used for cloning and transient overexpression. [file TPJ-97-391-s001.pdf]

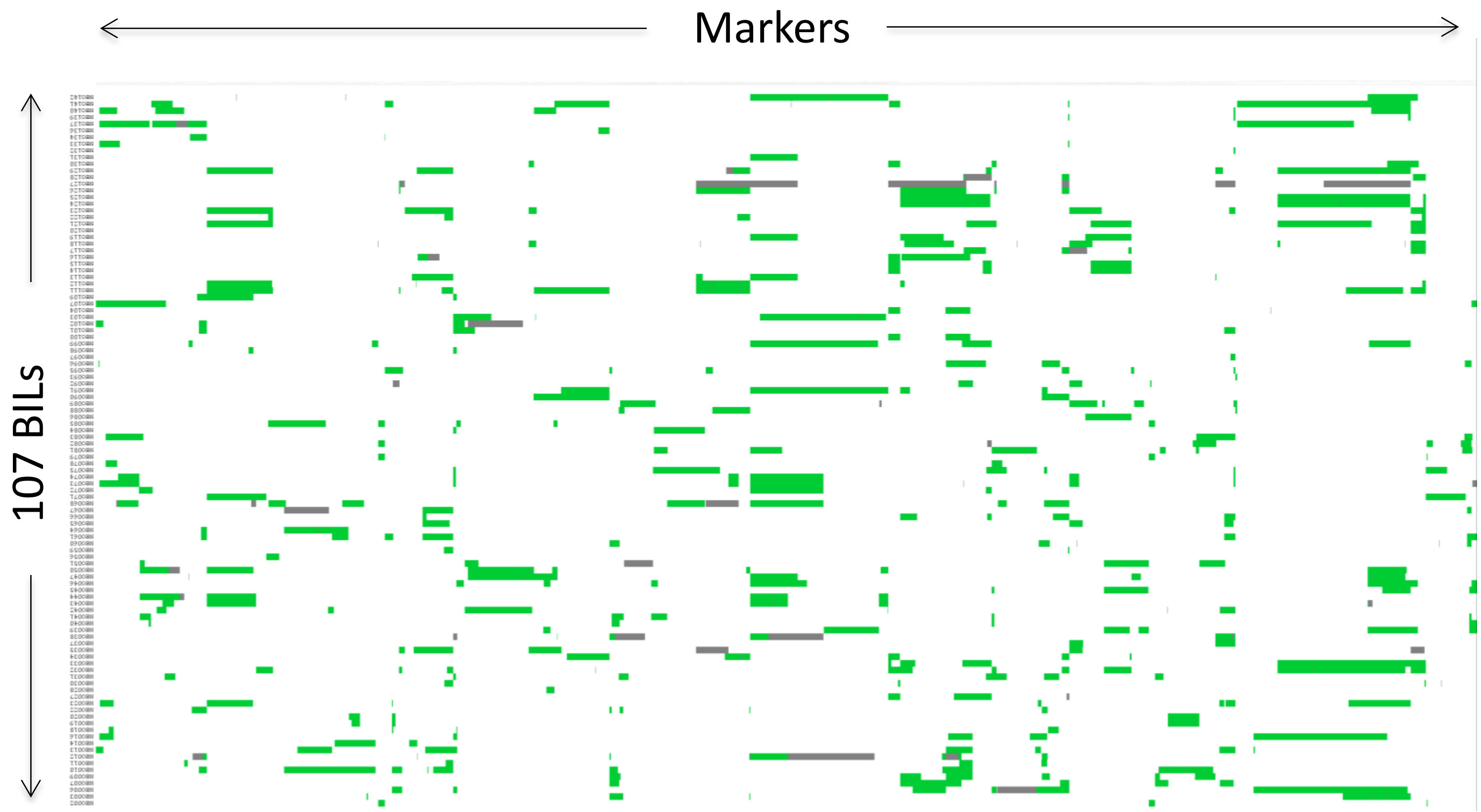

Figure S1.

Graphic representation of the 107 *S. neorickii* BILs. Polymorphisms that match *S. neorickii* are colored green, while polymorphisms matching TA209 are plotted in white, failed SNPs are colored in gray color . Detailed information is presented in Table S1

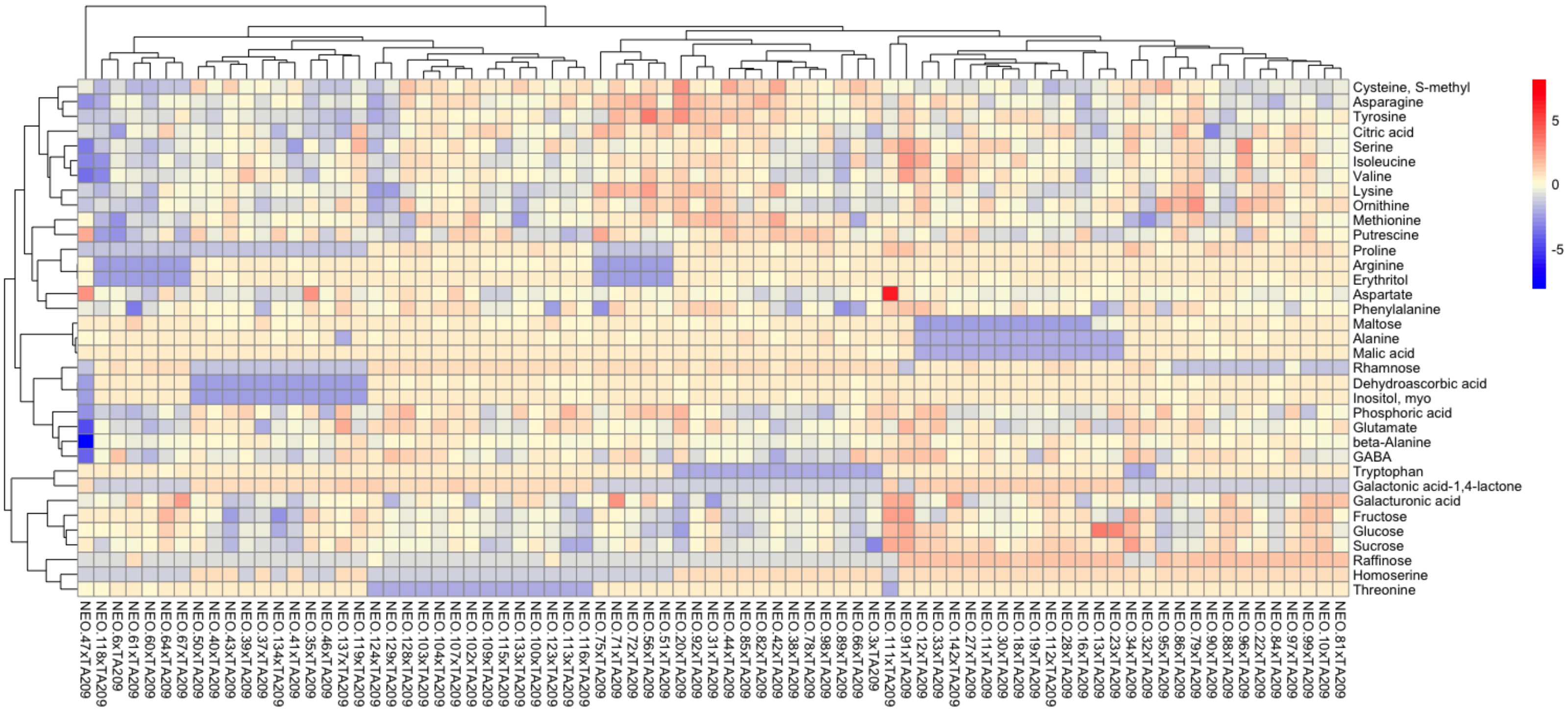

Figure S2.

Heat Map of the metabolites profiles of *S. neorickii* homozygous backcross inbred lines (BILs) heterozygous lines. Data are normalized to the mean response calculated for the TA209 line .The scale is logarithmic. Values presented are means of three replicates and are showed in false-color code.

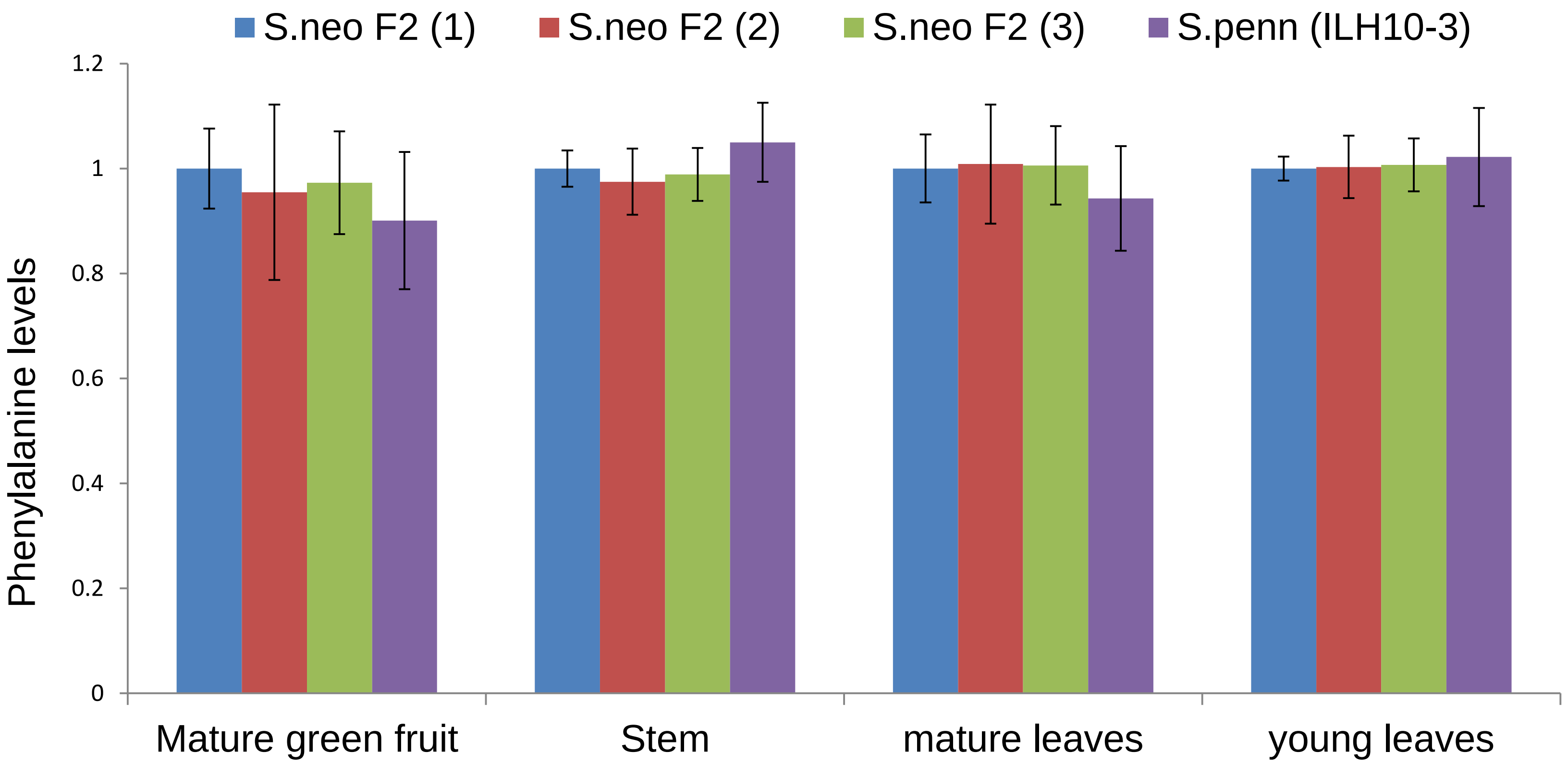

Figure S3.

Phenylalanine levels in mature green fruit, stem, mature and young leaves of F2 families from *S. neorickii*, as well as the *S. pennellii* line heterozygous (ILH10-3). Values are normalized to mean values of TA209. Values are means ± SE of three to eight replicates depending of the genotype. (1) Refers to material homozygous for the cultivated tomato allele, (2) the heterozygote and (3) represents material homozygous for the wild species allele.

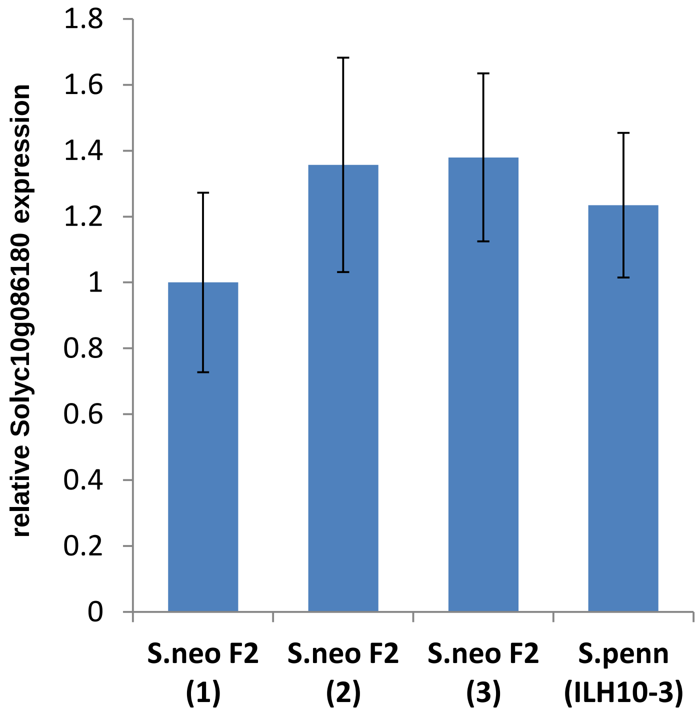

Figure S4.

Solyc10g086180 expression in mature green of F2 families from *S. neorickii*, as well as the *S. pennellii* line heterozygous (ILH10-3). Value are normalized to mean values of TA209. The abundance of PAL mRNA was measured by qRT-PCR. Values are normalized to mean values of TA209. Values are means ± Sdev of three to eight replicates depending of the genotype. (1) Refers to material homozygous for the cultivated tomato allele, (2) the heterozygote and (3) represents material homozygous for the wild species allele

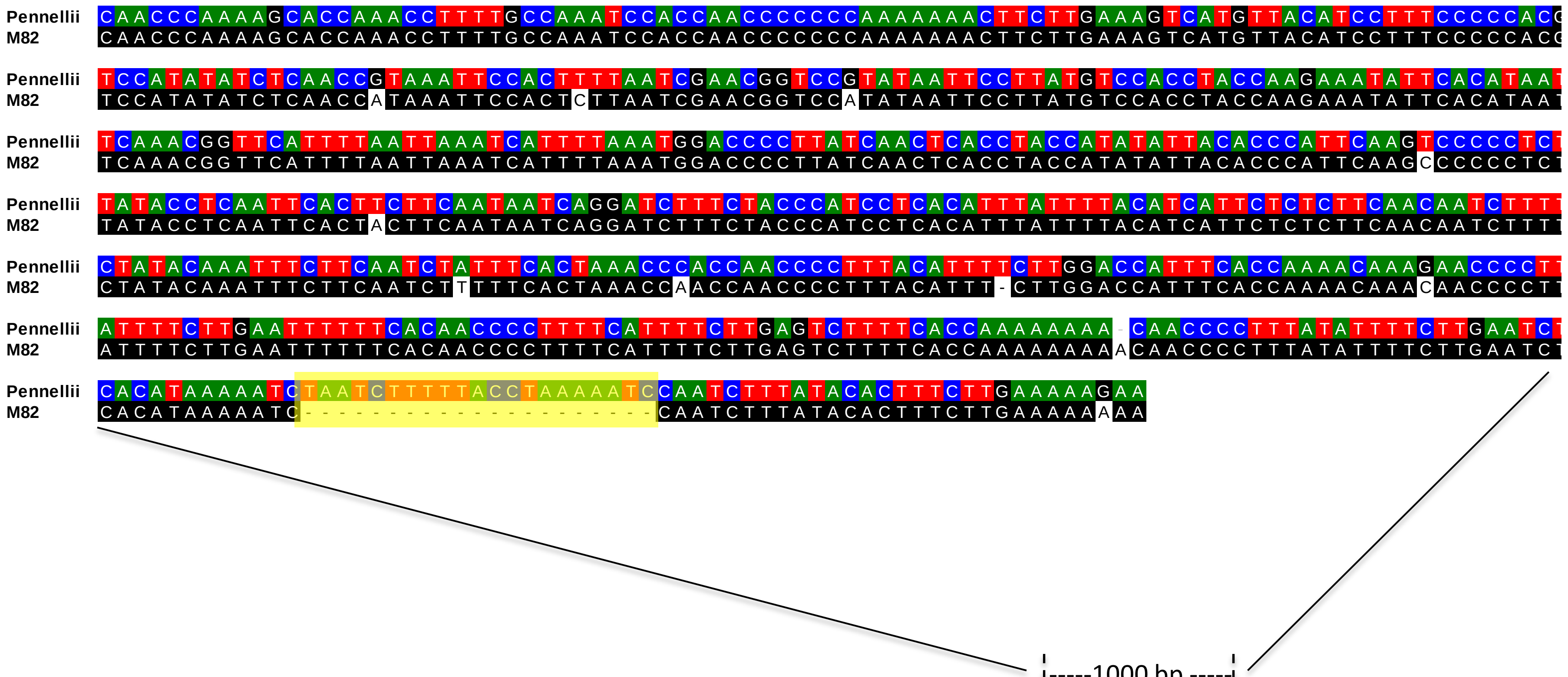

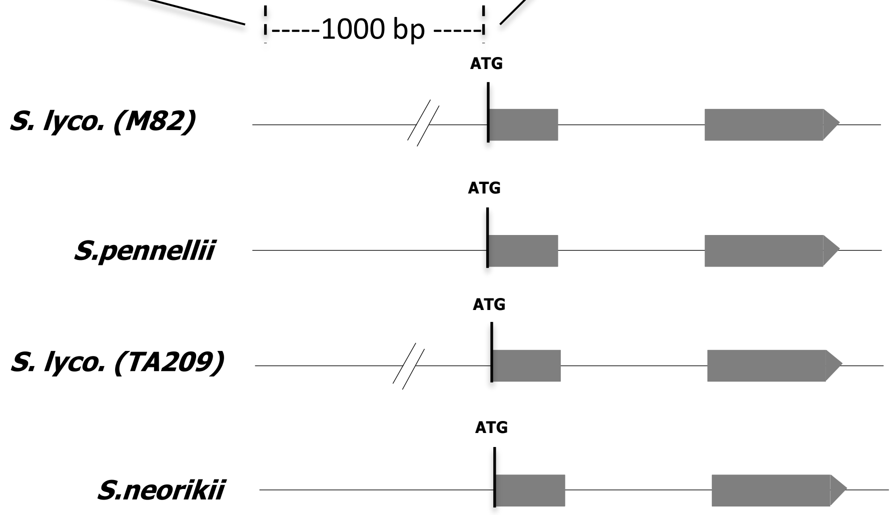

Figure S5.

Sequence analysis of 1000 bp upstream and open reading frame of M82 and *S. pennellii* for PAL gene (*Solyc10g086180),* same deletion was found for TA2009, while *S. neorickii* showed very similar to *S. pennellii* based on the draft genome sequences (http://solgenomics.net/)

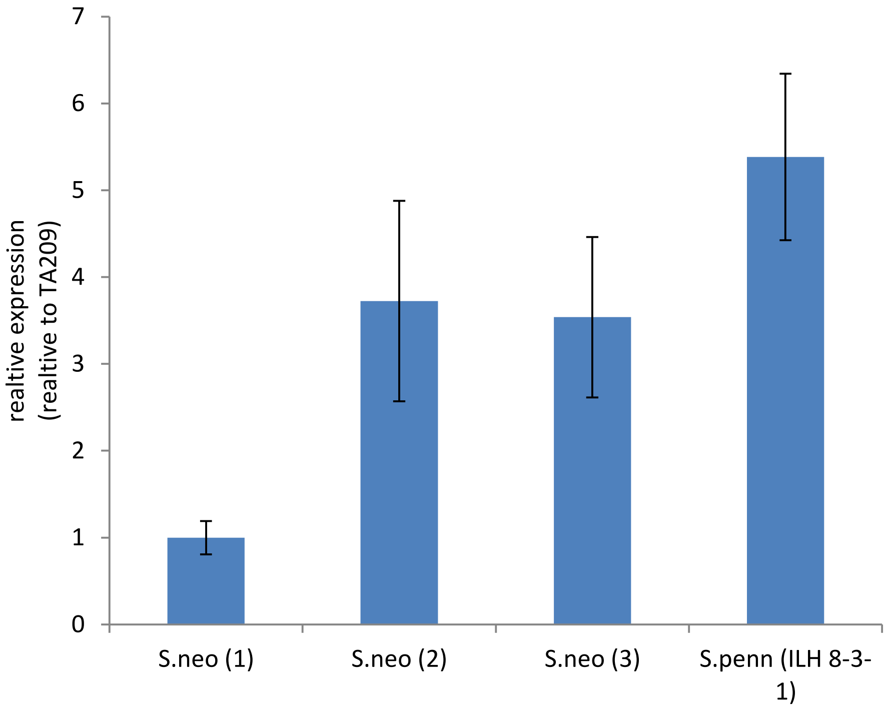

Figure S6*.*

Solyc08g083110 expression in mature green of F2 families from *S. neorickii*, as well as the *S. pennellii* line heterozygous (ILH8-3-1-3). The abundance of cystathionine gamma-lyase mRNAs was measured by qRT-PCR. Values are normalized to mean values of TA209. Values are means ± Sdev of three to eight replicates depending of the genotype. (1) Refers to material homozygous for the cultivated tomato allele, (2) the heterozygote and (3) represents material homozygous for the wild species allele.

```
Solyc08g083110      ATGTATCATAGACACAAGTATTCTACAACAAGTGCAATTTCTTTGGGCTAATATATACAG
Sopen08g031330      ------------------TATTCTAGAACAAATGCAATTTCTTTGGGCTAATATATACAG
                                      ******* ***** ****************************

Solyc08g083110      TAGCATTCTCCAATTAATTGACACATGTGTATATATTATGATATAAGATGAAATAAATTA
Sopen08g031330      TAGCATTCTCCAATTAATTGACACATGTG--TATATTATGATATAAGATGAAATAAATTA
                    *****************************  *****************************

Solyc08g083110      AAATAGCGTTGTTATTATTATTATTATTGAGTTTTAAAATAGCTTATTATTGTGGTGAGA
Sopen08g031330      AAATAGCG------TTATTATTATTATTGAGTTTTTAAATAGGTTATTATTGTGGTGAGA
                    ********      ********************* ****** *****************

Solyc08g083110      GGGTATACTCAATTGGTTGAGGCCCTCCATCTTTCATAGTGTAATTTTGGGTATATAT--
Sopen08g031330      GGGTATACTCAATTGGTTGAGGCCCTCCATCCTTCATAGTGTAATTTTGTATATATATAA
                    ******************************* *****************  *******  

Solyc08g083110      ATATATTACCTTTTGTGTACTTTTCCGATATCATTTCCCAAATACGTACTTTATCCAATA
Sopen08g031330      ATATATTACCTTTTGTGTACTTTTCCGATATCATTTCCCAACTACGTACTTTATCCAATA
                    ***************************************** ******************

Solyc08g083110      TAGTCGGAAGGGCGGGTACATTCGTTCTACCGATTCAATTTTGCTACTCACTACTCTATA
Sopen08g031330      TAGTCGGAAGGACGGGTACATTCGTTCTACCAATTCAATTTTGCTACTCACTACTCTATA
                    *********** ******************* ****************************

Solyc08g083110      ATATATAAACTAAGTCA------------------ATCCCTTTGTCCTAAAATATGTAAT
Sopen08g031330      ATGTATAAACT-AGTCAATAGCATTGACTATATACATACCTTTGTCCTAAAATATGTACT
                    ** ******** *****                  ** ******************** *

Solyc08g083110      ATATTATTTTTAATAAGAAGAATTATAATTGATAAGTATTTTCTATATAATTTTGAATAT
Sopen08g031330      CTATTATTTTTGATTATATGACTAATAATCTATAAGTATTTTCTATATAATTTTGAATAT
                     ********** ** * * ** * *****  *****************************

Solyc08g083110      TAAATTAATTTCATCTAAAAATTGATTTCCAAAAAAATAAAATAAACATATA--------
Sopen08g031330      TAAATTAATTTCATTTAGAGATTGATTTCCGAAAGAATAAAATAAACACATGTTTCCTCG
                    ************** ** * ********** *** ************* **        

Solyc08g083110      --GTTTTTATACATTTTGATTTGATACACCCGTTAATTATATGATAAATAAAATTATAAT
Sopen08g031330      TTATTTTTATACATTTTGATTTAGTACACCCCTTAATTATATGATAAATAAATTTATAAT
                       *******************  ******* ******************** *******

Solyc08g083110      TTTACTATATCCCTCTTAATGTAATAATTTTCTTTCGGCTTTGCGATAATAAGTTATTTA
Sopen08g031330      TTTACTATATCCCTCTTAATGTAATAATTTTCTTTCGGCTTTGTGATCATAAGTTATTTA
                    ******************************************* *** ************

Solyc08g083110      TATGTAATTGGAAATAATTAAACCATTGATAGCAATG--------ATAAAATAAAGATAT
Sopen08g031330      TATGTAATTGGAAATAATTAAACCATTGATAGCAATGATAAAATAATAAAATAAATATAT
                    *************************************        ********** ****

Solyc08g083110      GGCAGAGAGAGTATATTAGAACGGAGAGTATATTGATATAAATATTCTTTGTTTGGTCAA
Sopen08g031330      GGC--AGAGAGTATTTTAGAACGGAGAGTATATTAATATAAATATTCTTTGTT-------
                    ***  ********* ******************* ******************       

Solyc08g083110      TGGTCAATGGTCAATGTGAAAAAATACGTTTGTGTTGGTCCAATGTAAATTGAAGGTTAC
Sopen08g031330      TGGTCAATGGTCAATGTGAAAAAATACG-TTGTGTTGGTCCAATGTAAATTGAACGTTAC
                    **************************** ************************** *****

Solyc08g083110      ATAATTAGTGGCAAATTCCTTTAATGATCTCACTCCATTCTTATCTGTAAGTTTGCTGAA
Sopen08g031330      ATAATTAGTGGCAATTTCCTTTAATGATCTCACTCCATTCTTATCTGTAAGTTTGCTGAA
                    ************** *********************************************

Solyc08g083110      AATGAATCACAAGTGAAAAAGAAATAAAATAATTACTCTTGCCAATATTATTTTAATTGT
Sopen08g031330      AATGAA-CACAAGTGAAAAAGAAATAAAATAATTGCTCTAGCCAATATTATTTTAATTGT
                    ****** *************************** **** ********************

Solyc08g083110      AATTAATTAATTAATTATTGGTATTGGTTTAATTAATTATTTTCTATTATATATAGAGTG
Sopen08g031330      AATTTGGCATCCTAATATTGGTATTGGTTTAATTAATTATTTTCTATTATATATAGAGTG
                    ****    *    * *********************************************

Solyc08g083110      GTCTGTCCCCTTATTTTT
Sopen08g031330      GTCTGTCCCCTTATTTTT
                    ******************
```

Figure S7.

Sequence analysis of 1000 bp upstream and open reading frame of M82 and *S. pennellii* for cystathionine gamma lyase gene (*Solyc08g083110), a* similar deletion was found for TA2009, while *S. neorikii* showed very similar sequence to *S. pennellii* based on the draft genome sequences (http://solgenomics.net/)

```
Solyc08g083110      MADTLNQNTFSNKKRSSGSDDCDHHDDTLFVSKKQSKSLVWEDPAAALANARHEFGEHGG
Sopen08g031330      MADTLNQNTLSNKKRSSGSDDCDHHDDSFFVSKKQSKSLVWEDPAAALANARHEFGEHGG
                    *********:*****************::*******************************

Solyc08g083110      VNMSIEASATFTVMEPETMRRMFAGELGPDRDFFIYSRHFNPTVLNLGRLMAALEGTEAA
Sopen08g031330      VNMSIEASATFTVMEPETMRRMFAGELGPDRDFFIYSRHFNPTVLNLGRLMAALEGTEAA
                    ************************************************************

Solyc08g083110      YCTASGMSAISSVMLQLCSSGGHVVASQTLYGGTHALLTHFLPRACNITTSFVDIRDLEM
Sopen08g031330      YCTASGMSAISSVMLQLCSSGGHVVASQTLYGGTHALLTHFLPRACNITTSFVDIRDLEM
                    ************************************************************

Solyc08g083110      VKEAIVEGRTNVLYFESISNPTLTVANIPELSRIAHEKGVTVVVDNTFAPLVLSPVKLGA
Sopen08g031330      VKEAIVEGRTNVLYFESMSNPTLTVANIPELSRIAHENGVTVVVDNTFAPMVLSPVKLGA
                    *****************:*******************:************:*********

Solyc08g083110      DVVVHSISKYISGAADIIAGAVCGPASLVNSMMDLHQGSLMLLGPTMNPKVAFELAERLP
Sopen08g031330      DVVVHSISKYISGAADIIAGAVCGPASLVNSMMDLHQGSLMLLGPTMNPKVAFELAERLP
                    ************************************************************

Solyc08g083110      HLGLRMKEHCKRALEYGTRMTKLGLKVMYPGLEDHPDHVLIKSMANKEYGYGGILCVDME
Sopen08g031330      HLGLRMKEHCKRALEYATRMTKLGLKVIYPGLEDHPDHVLIKSMANKEYGYGGILCVDME
                    ****************.**********:********************************

Solyc08g083110      TEERANRLMNVLQNFTQFGFMAVSLGYYETLMSCSGSSTSSELNNEEKELAGISPGLVRM
Sopen08g031330      TEERANRLMNVLQNFTQFGFMAVSLGYYETLMSCSGSSTSSELNNEEKELAGISPGLVRM
                    ************************************************************

Solyc08g083110      SIGYNGSLEQKWSQLDKALS----KMPF
Sopen08g031330      SIGYNGSLEQKWSQLDKALSKMQEKMPF
                    ********************    ****
```

Figure S8.

Protein sequence of the cystathionine gamma lyase (*Solyc08g083110)* gene of M82 and *S. pennellii*

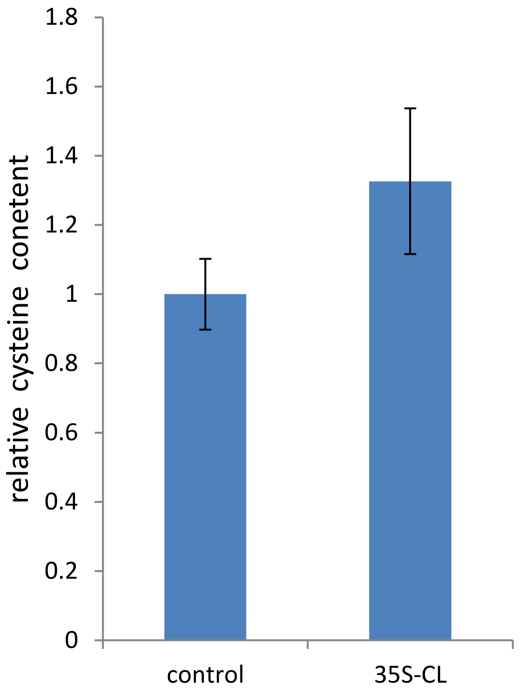

Figure S9.

Cysteine levels in transient overexpression of cystathionine gamma lyase (Solyc08g083110) gene and the control. Data are presented as means of five biological replicates ± Sdev

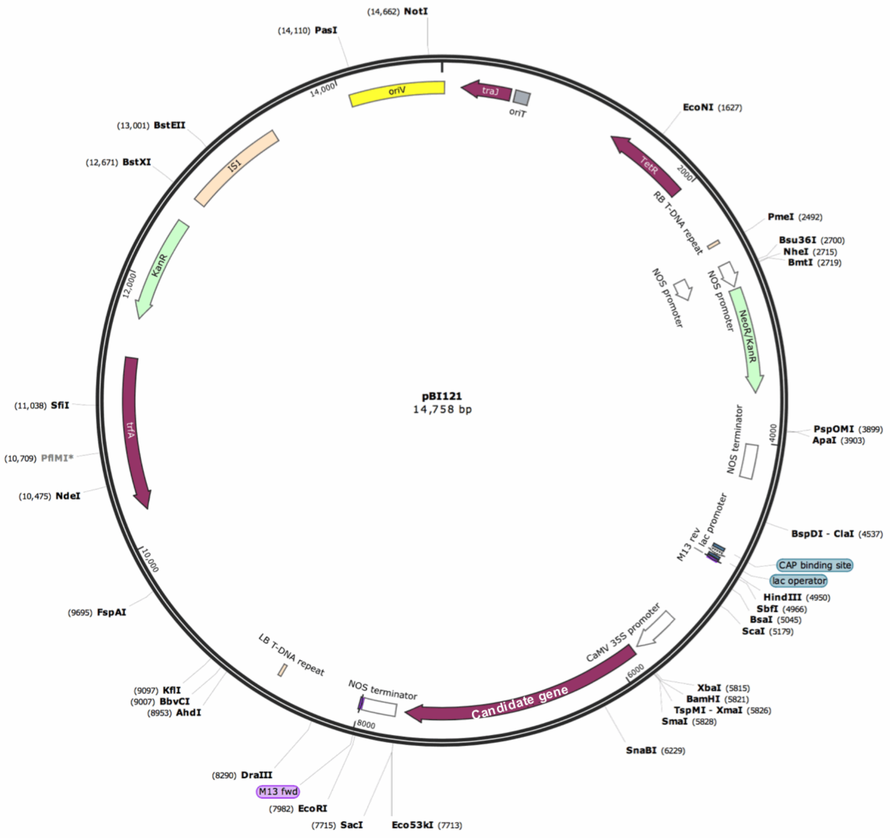

Figure S10.

Vector map used for cloning and transient overexpression
